# Mechanisms of chemotherapy failure in refractory/relapsed acute myeloid leukemia: the role of cytarabine resistance and mitochondrial metabolism

**DOI:** 10.1038/s41419-025-07653-6

**Published:** 2025-04-23

**Authors:** Soo Yeon Chae, Se-Young Jang, Jinhui Kim, Sehyun Hwang, Disha Malani, Olli Kallioniemi, Seung Gyu Yun, Jong-Seo Kim, Hugh I. Kim

**Affiliations:** 1 Department of Chemistry, Korea University, Seoul, Republic of Korea; 2 Center for Proteogenome Research, Korea University, Seoul, Republic of Korea; 3 School of Biological Sciences, Seoul National University, Seoul, Republic of Korea; 4 Department of Medical Oncology, Dana-Farber Cancer Institute, Boston, MA USA; 5 Institute for Molecular Medicine Finland, FIMM, University of Helsinki, Helsinki, Finland; 6 Science for Life Laboratory, Department of Oncology and Pathology, Karolinska Institute, Solna, Sweden; 7 Department of Laboratory Medicine, Korea University College of Medicine, Seoul, Korea; 8 Center for RNA Research, Institute of Basic Science, Seoul National University, Seoul, Korea

**Keywords:** Mechanisms of disease, Stress signalling, Acute myeloid leukaemia

## Abstract

Acute myeloid leukemia (AML) is an aggressive hematological malignancy. Patients with wild-type FLT3 relapsed or refractory (R/R) AML face significant therapeutic challenges due to the persistent lack of effective treatments. A comprehensive understanding of the mechanisms underlying chemotherapy resistance is needed to the development of effective treatment strategies. Therefore, we investigated the molecular mechanisms underlying cytarabine (Ara-C) resistance and daunorubicin (DNR) tolerance in Ara-C-resistant RHI-1 cells derived from the wild-type FLT3 AML cell line SHI-1. Quantitative analysis of intracellular drug concentrations, proteomics, and phosphoproteomics showed that DNR resistance in Ara-C-resistant RHI-1 cells is driven by metabolic remodeling toward mitochondrial metabolism, upregulation of DNA repair pathways, and enhanced reactive oxygen species (ROS) detoxification rather than reduced drug uptake. Moreover, targeting these compensatory mechanisms, particularly the OXPHOS complex I proteins, significantly improved the efficacy of both Ara-C and DNR. Conclusively, these findings highlight mitochondrial metabolism and DNA repair as critical factors in chemotherapy resistance and offer valuable insights into potential therapeutic targets for enhancing treatment outcomes in patients with wild-type FLT3 R/R AML.

## Introduction

Acute myeloid leukemia (AML) is an aggressive hematological malignancy characterized by immature differentiation and abnormal proliferation [1]. Since the discovery of the potent cytotoxicity of cytarabine (Ara-C), the high-dose Ara-C regimen (HiDAC) and the 7 + 3 regimen, which consists of Ara-C and daunorubicin (DNR) (three once-daily injections of DNR with continuous 7-day infusion of Ara-C), have been widely used as standard treatments for AML [2]. However, ~10–40% of patients with AML under the age of 60 and 40–60% of those over 60 years experience refractory or relapsed AML (R/R AML) [1]. Targeted therapies, such as the administration of FLT3 inhibitors, have been used for patients with FLT3-ITD mutations [3]. Although these targeted therapies improved response rates and survival outcomes in FLT3-ITD mutated R/R AML, patients with wild-type FLT3 R/R AML remain therapeutically challenged due to drug resistance. Despite the failure of Ara-C- or DNR-based first-line chemotherapy, it is still being used as a second-line therapy for some patients with wild-type FLT3 R/R AML. For instance, ADE (Ara-C, DNR, etoposide) [4], 5 + 2 regimen (Ara-C and DNR) [5], and 7 + 3 regimen are used as second-line chemotherapy for patients with wild-type FLT3. Expectedly, approximately 40–50% of patients receiving Ara-C- or DNR-based second-line chemotherapy fail to achieve complete remission. Therefore, better insights are needed to enhance the treatment outcomes of patients with wild-type FLT3 R/R AML.

A comprehensive understanding of differentially regulated biological pathways and the mechanism of chemotherapy failure is necessary to improve chemotherapy strategies for FLT3-ITD wild-type R/R AML. However, current research has focused mainly on risk classification and signatures for predicting treatment responses from omics data.

Therefore, this study aimed to investigate the mechanisms underlying chemotherapy failure in Ara-C-resistant RHI-1 cells derived from the wild-type FLT3 AML cell line SHI-1. Moreover, we identified the effective target to enhance the efficacy of Ara-C and DNR in RHI-1 and patient-derived cells. This study offers critical insights into the molecular basis of treatment failure in wild-type FLT3 R/R AML and proposes novel strategies to overcome resistance.

## Results

### Drug tolerance of Ara-C-resistant AML cells

SHI-1 cells have been characterized as a wild-type FLT3 AML cell line in various studies [6–8]. Additionally, FLT3-ITD mutation fragment analysis confirmed that SHI-1 and RHI-1 cells were wild-type FLT3 AML cells (Fig. S1A, B). Ara-C resistance was induced via long-term subculturing of SHI-1 cells under Ara-C treatment, as previously described [8]. Cell viability assay was conducted to elucidate the responses of SHI-1 and RHI-1 cells to Ara-C (0–0.64 μM) and DNR (0–2 μM) either alone or as a combination after 24 and 48 h of treatment (Tables S1, 2). The various drug treatments and associated terms used in this study are listed in Table 1.

**Table 1:** Comprehensive list of drug combinations and associated terms.

		Ara-C	Ara-C	Ara-C	Ara-C
	μM	0	0.16	0.32	0.64
DNR	0	—	A0.16	A0.32	A0.64
DNR	0.08	D0.08	C(A0.16 + D0.08)	C(A0.32 + D0.08)	C(A0.64 + D0.08)
DNR	0.16	D0.16	C(A0.16 + D0.16)	C(A0.32 + D0.16)	C(A0.64 + D0.16)
DNR	0.2	D0.2	C(A0.16 + D0.2)	C(A0.32 + D0.2)	C(A0.64 + D0.2)
DNR	0.3	D0.3	C(A0.16 + D0.3)	C(A0.32 + D0.3)	C(A0.64 + D0.3)
DNR	0.61	D0.61	C(A0.16 + D0.61)	C(A0.32 + D0.61)	C(A0.64 + D0.61)
DNR	2	D2	C(A0.16 + D2)	C(A0.32 + D2)	C(A0.64 + D2)

Ara-C, DNR, and combination treatment are denoted by “A,” “D,” and “C” respectively.

**Fig. 1:**
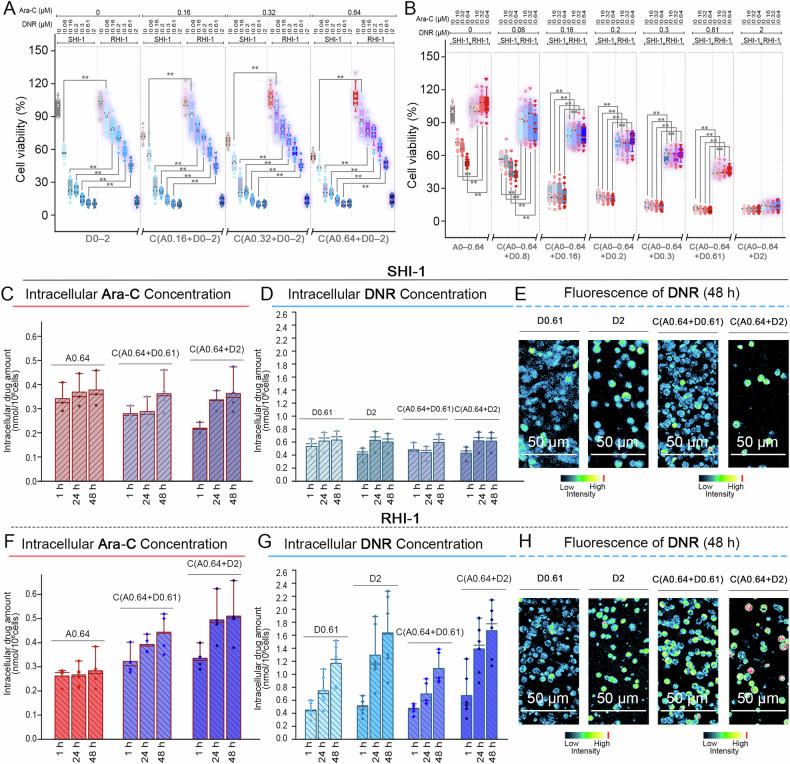
Impact of Ara-C and DNR combinations on cell viability and intracellular drug accumulation in SHI-1 and RHI-1 cell lines. **A** Box plots showing the viabilities of SHI-1 and RHI-1 cells in the D0–D2, C(A0.16 + D0–2), C(A0.32 + D0–2), and C(A0.64 + D0–2) groups at 48 h. **B** Box plots showing the viabilities of SHI-1 and RHI-1 cells in the A0–A0.64, C(A0–A0.64 + D0.08), C(A0–A0.64 + D0.16), C(A0–A0.64 + D0.2), C(A0–A0.64 + D0.3), C(A0–A0.64 + D0.61), and C(A0–A0.64 + D0.2) groups at 48 h. **C**, **D**, **F**, **G** Intracellular drug concentration in SHI-1 and RHI-1 cells. Bar charts with data points of intracellular Ara-C concentrations in SHI-1 (**C**) and RHI-1 (**F**) cells in the A0.64, C(A0.64 + D0.61), and C(A0.64 + D2) groups. Bar charts with data points of intracellular DNR concentrations in SHI-1 (**D**) and RHI-1 (**G**) cells in the D0.61, D2, C(A0.64 + D0.61), and C(A0.64 + D2) groups. **E**, **H** Confocal images of intracellular DNR in SHI-1 (**E**) and RHI-1 (**H**) cells in the D0.61, D2, C(A0.64 + D0.61), and C(A0.64 + D2) groups. (*n* ≥ 9, ∗*p* < 0.05, ∗∗*p* < 0.01, ∗∗∗*p* < 0.001, ∗∗∗∗*p* < 0.0001).

Ara-C and DNR treatments significantly decreased the viability of SHI-1 cells in a concentration-dependent manner within 48 h (Fig. 1A, B). The cell viability results for the combination treatments followed a similar pattern to that of the single treatments with DNR. Although the combination treatment decreased cell viability to the same extent as DNR monotherapy, no additional synergistic effects were detected (Supplementary Discussion 1).

Compared to SHI-1 cells, RHI-1 cells exhibited resistance to Ara-C and DNR (*p* < 0.01) treatments (Fig. 1A, B). Although DNR treatment was cytotoxic to RHI-1 cells, its effect was 1.3–4 times lower than that in SHI-1 cells. Despite the different mechanisms of action of DNR and Ara-C, RHI-1 cells exhibited significant tolerance to DNR, regardless of the presence or absence of Ara-C. Collectively, RHI-1 cells were resistant to both Ara-C and DNR and the combined treatments (Supplementary Discussion 1).

### Intracellular drug concentrations in SHI-1/RHI-1 cells

Considering that the cytotoxicity of Ara-C and DNR is related to the intracellular concentrations of the drugs, we determined the concentrations of both drugs ([Drug]_cell_) in SHI-1 and RHI-1 cells. For these analyses, we used 0.64 μM Ara-C and 0.61, 2 μM DNR (Supplementary Discussion 2).

In SHI-1 cells, [Ara-C]_cell_ levels steadily increased over 48 h in all treatment groups, though the rate of increase was slightly attenuated in the DNR-treated groups (Fig. 1C and Table S3). In contrast, RHI-1 cells showed only modest changes over time in the A0.64 group, while the addition of DNR led to a more pronounced, time-dependent increase in [Ara-C]_cell_ levels (Fig. 1F and Supplementary Discussion 2). Although Ara-C uptake was higher in RHI-1 cells than in SHI-1 cells under the combined treatments, it did not induce additional cytotoxicity in RHI-1 cells, suggesting that RHI-1 cells may have developed Ara-C resistance by inhibiting the mechanism of action of Ara-C.

Furthermore, The [DNR]_cell_ values of SHI-1 cells in the D0.61, D2, C(A0.64 + D0.61), and C(A0.64 + D2) groups showed a similar trend, ranging from 0.5 to 0.6 nmol/10^6^ cells (Fig. 1D and Supplementary Discussion 2). The [DNR]_cell_ values of RHI-1 cells in the D0.61, D2, C(A0.64 + D0.61), and C(A0.64 + D2) groups were higher than those of SHI-1 cells by 1.8, 2.7, 1.8, and 2.7 times, respectively (Fig. 1G). Conversely, RHI-1 cells demonstrated tolerance to DNR, suggesting their ability to survive at higher drug concentrations (Supplementary Discussion 2).

Using confocal fluorescence microscopy, we measured DNR fluorescence intensity (Supplementary Discussion 2) and found that RHI-1 cells consistently displayed stronger fluorescence than SHI-1 cells (Fig. 1E, H). Overall, these results suggest that Ara-C resistance in RHI-1 may reduce the cytotoxicity of DNR by interrupting the cytotoxic mechanisms of DNR.

### Quantitative proteomic/phosphoproteomic profiling resistance-associated protein signature

Protein phosphorylation plays a pivotal role as a primary effector in various cellular processes, and system-wide profiling of phosphorylated effectors provides information on active signaling pathways. To investigate the signaling pathways associated with Ara-C resistance-induced DNR tolerance in RHI-1, we performed quantitative global proteomics and phosphoproteomics using highly multiplexed isobaric labeling and MS3-based accurate quantification (Fig. S2A, B and Supplementary Data Files 1 and 2).

Protein expression and phosphorylation levels in all groups, including untreated SHI-1, as well as in RHI-1 under untreated or treated conditions (D0.61, D2, C(A0.64 + D0.61), or C(A0.64 + D2)), are shown in each heatmap (Fig. S3A, B). The heatmaps of untreated RHI-1 groups exhibited similar patterns as those of the untreated SHI-1 group. In contrast, these were significantly different from the other drug-treated conditions. Moreover, the column correlation values for untreated SHI-1 and RHI-1 groups were ~0.7 or higher, whereas they were markedly below –0.3 when compared with the other drug-treated groups (Fig. S3C, D). These trends were also reflected in the principal component analysis (PCA), wherein the PC1 and PC2 axes for protein expression (Fig. S3E) and phosphorylation levels (Fig. S3F) of the untreated SHI-1 group closely matched those of the RHI-1 group. Similarly, the PCA values in these groups were notably different from those in the other conditions. These results indicate that although long-term sub-culturing of SHI-1 cells was required for generating the RHI-1 cell line, this process did not significantly affect the differences in protein expression between SHI-1 and RHI-1 cells compared with the drug exposure, which exerted pronounced effects.

Protein expression and phosphorylation levels in the D0.61- and D2-treated RHI-1 groups, shown as heatmaps, closely matched those of the C(A0.64 + D0.61) and C(A0.64 + D2) groups, respectively. The values of column correlation for these two pairs were approximately 0.5 or higher, and PC1 and PC2 were highly similar to each other. It is suggested that the activation of Ara-C is inhibited in RHI-1 cells (Supplementary Discussion 3), which results in no observed additive or synergistic effect between Ara-C and DNR.

For simplifying the analysis, we focused on the untreated and C(A0.64 + D0.61)- and C(A0.64 + D2)-treated RHI-1 groups rather than on the D0.61- and D2-treated RHI-1 groups for further investigation.

**Fig. 2:**
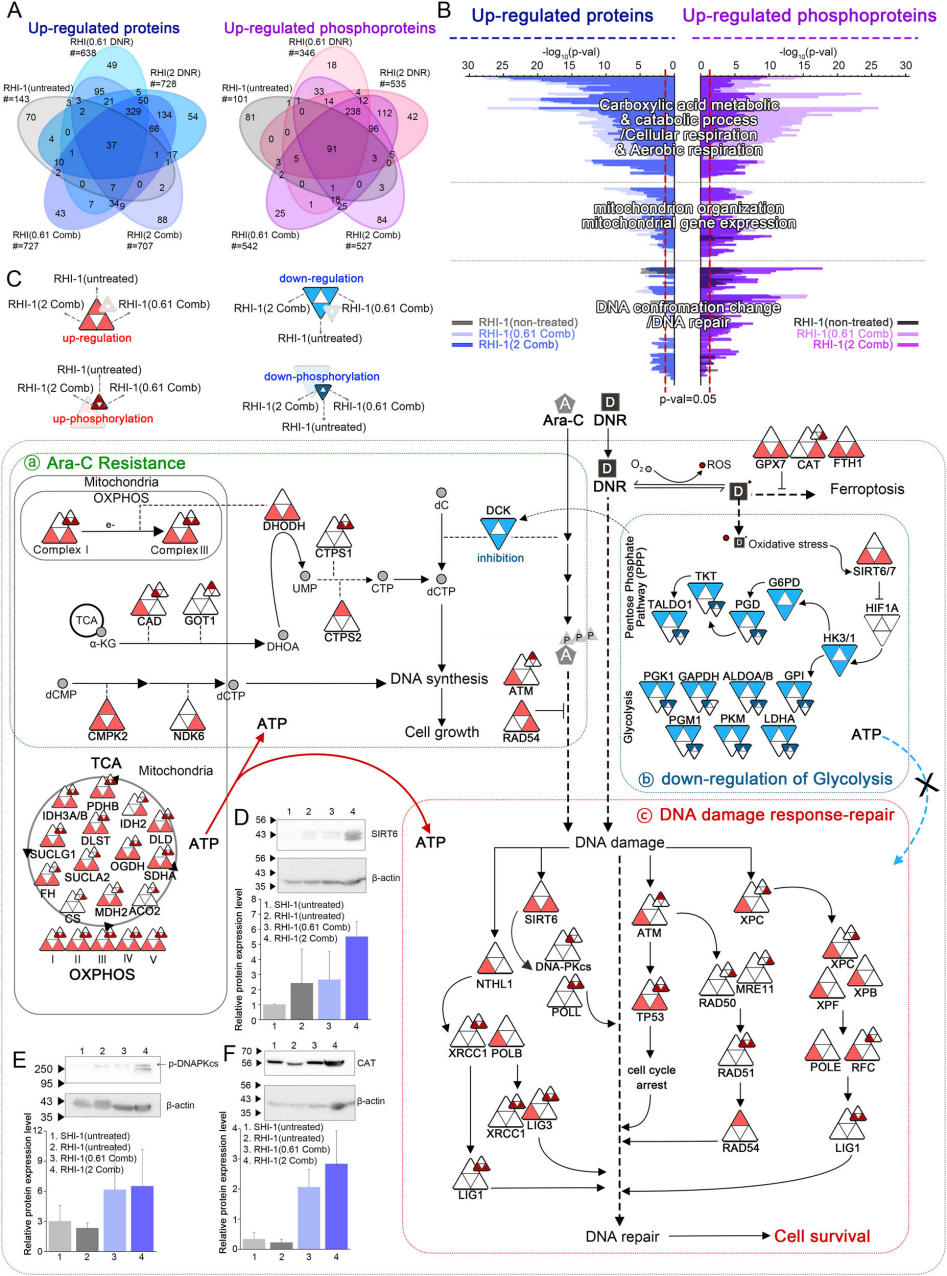
Proteomic and phosphoproteomic analysis of cellular pathways involved in Ara-C and DNR resistance in RHI-1 cells. **A** Venn diagram showing relationships between upregulated proteins and upregulated phosphoproteins. **B** Bar-charts showing the overlap between significantly enriched gene ontology biological processes (GOBPs) by differentially expressed proteins (DEPs) and phosphor-DEPs. **C** Network model describing the cellular pathways of upregulated/downregulated proteins or phosphorylation in RHI-1 cells. The diagram shows the regulation and phosphorylation status of protein under three conditions: RHI-1(untreated), RHI-1(0.61 Comb), and RHI-1(2 Comb). The large triangles represent protein expression levels, and the small triangles represent phosphorylation levels. (Red-colored large triangles indicate up-regulation, and red-colored small triangles indicate up-phosphorylation. Blue-colored one is down-regulation. Ara-C and DNR are denoted by “A” and “D”, respectively. **D**–**F** Quantification of the levels of SIRT6 (**D**), phosphor-DNAPKcs (**E**) and CAT (**F**) using immunoblotting. Bar charts represent mean ± standard deviation (S.D.) of values derived from three independent experiments.

To elucidate the mechanism of chemotherapy resistance, we performed GO enrichment analysis of upregulated Differentially expressed proteins (DEPs) and phospho-proteins (phos-DEPs) (Fig. 2B, Supplementary Discussion 3, and Supplementary Data File 5) using DAVID [9]. Upregulated DEPs and phos-DEPs in the RHI-1(untreated) group were mainly enriched in processes associated with DNA repair, including DNA metabolic process, regulation of cell cycle, and DSB repair. Additionally, upregulated DEPs and phos-DEPs in the RHI-1(0.61 Comb) and RHI-1(2 Comb) groups were enriched in biological processes associated with mitochondrial metabolism, including carboxylic acid metabolism, catabolic processes, cellular respiration, aerobic respiration, mitochondrion organization, and mitochondrial gene expression. Moreover, upregulated DEPs and phos-DEPs in the RHI-1(2 Comb) group were mainly enriched in DNA conformational changes and DNA repair.

To understand the combined effects, we constructed a network model based on GO enrichment analysis (Figs. 2C, and S4A–C, Supplementary Discussion 4, Table S4), Kyoto Encyclopedia of Genes and Genomes (KEGG) pathway analysis [10], and previously reported signaling pathways. As shown in Fig. 2C, DCK, a key enzyme in the nucleotide salvage pathway, was downregulated in the RHI-1(untreated) group. DCK downregulation affects the pyrimidine salvage pathway and inhibits Ara-C activation. To maintain the nucleotide pools, de novo pyrimidine biosynthesis, which synthesizes pyrimidine nucleotides from other nucleotide precursors, is activated and upregulated in RHI-1 cells [11, 12] (Fig. 2C-a). This synthesis caused a shift in metabolic preference toward mitochondrial metabolism over glycolysis in RHI-1 cells, as indicated by the downregulation of glycolytic proteins and upregulation of mitochondrial proteins (Fig. 2C-b). Additionally, a network model showed that upregulation of the ATM-RAD54-mediated DNA repair pathway led to cell survival in the RHI-1(untreated) group [13, 14].

DNR induces DNA damage, cell death, ferroptosis, and reactive oxygen species (ROS) generation, leading to oxidative stress, glycolysis inhibition, and mitochondrial dysfunction. Glycolysis inhibition in synergy with mitochondrial dysfunction induces ATP depletion and represses the synthesis of cellular building blocks [15–22]. However, our network model indicated that drug tolerance-related cell survival was driven by DNA repair, ROS detoxification, and metabolic remodeling in the RHI-1(0.61 Comb) and RHI-1(2 Comb) groups. As shown in Fig. 2C-c, the already activated/upregulated ATM rapidly recognized damaged DNA and subsequently activated/upregulated TP53 proteins to arrest the cell cycle and upregulated RAD50, MRE11, RAD51, and RAD54 proteins for DNA repair. XPC, SIRT6, and NTHL1 further recognized damaged DNA and activated/upregulated repair proteins, including XPF, XPB, POLE, RFC, LIG1, DNA-PKcs, POLL, XRCC1, POLB, and LIG3 [23, 24]. Additionally, the network model showed that ROS detoxification proteins such as CAT, GPX7, and FTH1 were activated/upregulated in the RHI-1(0.61 Comb) and RHI-1(2 Comb) groups [21, 25–27]. These proteins inhibited ferroptosis and mitochondrial dysfunction, leading to the upregulation of mitochondrial metabolism in the RHI-1(0.61 Comb) and RHI-1(2 Comb) groups. Based on these results, it can be speculated that RHI-1 cells received an adequate supply of ATP and dNTP due to mitochondrial metabolism, which enhanced Ara-C resistance, DNR tolerance, and cell survival.

To validate these findings, we performed immunoblotting for three representative upregulated proteins (Fig. 2D–F): CAT for the antioxidant process, SIRT6 for DNA repair and glycolysis, and phos-DNAPKcs for DNA repair. Consistent with the network model, CAT, SIRT6, and phos-DNAPKcs were upregulated in the RHI-1(0.61 Comb) and RHI-1(2 Comb) groups.

Furthermore, we conducted GO analysis using previously reported global proteomic and phosphoproteomic data from FLT3 wild-type R/R AML [28], pediatric/adult R/R AML [29], and FLT3 wild-type [30] patient-derived samples (Supplementary Data File 6 and Fig. S4D–G). Similar to our network model, upregulated DEPs and phos-DEPs in these data were enriched in DNA repair, ROS detoxification, and mitochondrial metabolism GOBP. Collectively, these results suggest that ROS detoxification, mitochondrial metabolism, and DNA repair contribute to DNR tolerance in RHI-1 cells and in previously reported FLT3 wild-type [28], pediatric/adult [29], and FLT3 wild-type R/R AML patient cells.

### The role of mitochondrial metabolism: Inhibition of OXPHOS and DNA repair

To verify the inhibition of ATP depletion, we measured the cellular ATP levels in SHI-1 and RHI-1 cells in the control (untreated), A0.64, C(A0.64 + D0.61), and C(A0.64 + D2) groups at 48 h (Fig. S5). ATP levels were higher in RHI-cells than in SHI-1 cells by 1.13-, 2.04-, 1.45-, and 3.38-fold in the untreated-, A0.64, C(A0.64 + D0.61), and C(A0.64 + D2) groups, respectively, suggesting that ATP production was not suppressed in drug-treated RHI-1 cells.

**Fig. 3:**
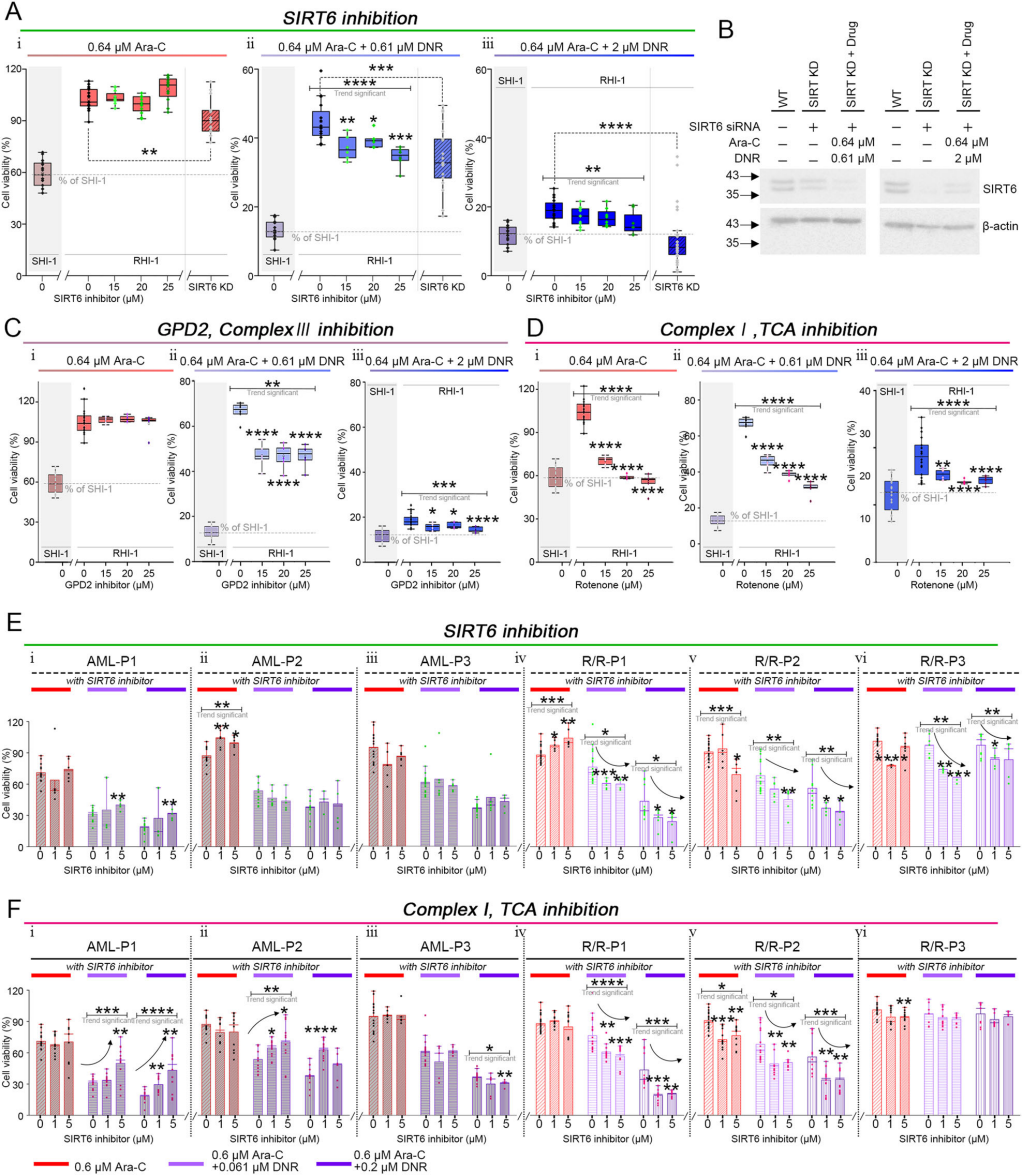
Inhibition of SIRT6, GPD2, and mitochondrial complex I reduces AML cell and patient sample viability under Ara C and DNR treatment. **A** Box plots of cell viability of under (i) 0.64 μM Ara-C, (ii) 0.61 μM, and (iii) 2 μM DNR with SIRT6 inhibitor. **B** Western blot analysis confirming siRNA-mediated knockdown of SIRT6 in RHI-1 cells. Cellular ATP levels in SHI-1 and RHI-1 cells under drug treatment. **C** Box plots of cell viability under (i) 0.64 μM Ara-C, (ii) 0.61 μM, and (iii)) 2 μM DNR with GPD2 inhibitor and **D** rotenone. **E** Box plots of cell viability of patient samples under (i) 0.64 μM Ara-C, (ii) 0.61 μM, and (iii) 2 μM DNR with SIRT6 inhibitor and **F** rotenone. Box plots represent mean ± standard deviation (S.D.) of values derived from over six independent experiments.

SIRT6 inhibits glycolysis, enhances mitochondrial metabolism [31–33], and mainly regulates DNA-PKC-mediated DNA repair (Fig. 2C) [31, 32, 34]. To investigate the role of mitochondrial metabolism and DNA repair in DNR tolerance, drug treated-RHI-1 cells were treated with 0 (without), 15, 20, and 25 μM of SIRT6 inhibitor (Fig. 3A). SIRT6 inhibitor did not significantly affect the viabilities of RHI-1 cells in the A0.64 group (Fig. 3A, i). In contrast, the viabilities of RHI-1 cells in the C(A0.64 + D0.61) and C(A0.64 + D2) groups decreased with increasing concentrations of SIRT6 inhibitor (Fig. 3A, ii-iii).

Similar to the treatment with a SIRT6 inhibitor, siRNA-mediated knockdown of Sirt6 in RHI-1 (SIRT6 KD-RHI-1) cells resulted in a significant decrease in the viability of drug-treated cells compared with that of wild-type RHI-1 cells. SIRT6 knockdown was achieved by transfecting RHI-1 cells with a SIRT6-specific siRNA using a lipid-based transfection reagent. The efficacy of SIRT6 transfection was validated using immunoblotting (Fig. 3B), confirming the knockdown of SIRT6. The values for viability of SIRT6 KD-RHI-1 cells were 89, 34, and 10 under A0.64-, C(A0.64 + D0.61)-, and C(A0.64 + D2)-treated conditions, respectively (Fig. 3A; “SIRT6 KD”).

Overall, these findings suggest that inhibition of DNA repair, as well as the plasticity of cell metabolism through SIRT6 inhibition, can reduce the survival rate of DNR-treated RHI-1 cells.

Furthermore, mitochondrial metabolism was inhibited by targeting OXPHOS proteins. We initially suppressed glycerol-3-phosphate dehydrogenase (GPD2), which is upstream of the OXPHOS complex III, using iGP-1 (Fig. 3C). Drug-treated-RHI-1 cells were treated with 0, 15, 20, and 25 μM of iGP-1, followed by cell viability assessment after 48 h. iGP-1 treatment was ineffective in RHI-1 cells in the A0.64 group (Fig. 3C, i). This is because GPD2 inhibition does not affect DHODH function—an enzyme important for Ara-C resistance. Moreover, RHI-1 cells in the A0.64-treated group did not mainly depend on mitochondrial metabolism for ATP generation. In contrast, cells in the C(A0.64 + D0.61) and C(A0.64 + D2) groups were dependent on mitochondrial metabolism. Hence, iGP-1 treatment effectively reduced the viability of RHI-1 cells in the C(A0.64 + D0.61)- and C(A0.64 + D2) groups.

Additionally, OXPHOS complex I was suppressed using rotenone [35] (Fig. 3D). Rotenone treatment decreased the viabilities of RHI-1 cells in the A0.64 group in a concentration-dependent manner (Fig. 3D, i). Also, rotenone treatment (0–25 μM) reduced the viabilities of RHI-1 cells in the C(A0.64 + D0.61) and C(A0.64 + D2) from 67–31% to 19–15%, respectively (Fig. 3C, ii and iii). Treatment with 25 μM of rotenone reduced the viability of RHI-1 cells to values observed for SHI-1 cells in the A0.64, C(A0.64 + D0.61), and C(A0.64 + D2) groups. Inhibition of OXPHOS complex I causes broad suppression of the entire OXPHOS pathway, impairing the function of DHODH, a key protein involved in Ara-C resistance. Conclusively, these results indicate that inhibiting OXPHOS complex 1 is the most effective for treating RHI-1 cells.

### Inhibition of OXPHOS and DNA repair in AML patients

We conducted in vitro drug sensitivity tests using six patient samples—three from the treatment-responsive AML group (AML-P1, P2, and P3) and three from the relapsed/refractory (R/R) AML group (R/R-P1, P2, and P3) (Table S5). Maximum plasma concentrations of Ara-C and DNR, calculated using MWpharm + +, were determined to be 0.6 μM for Ara-C and 0.061 μM for DNR. Patient-derived cells, categorized into the A0.6, C(A0.6 + D0.061), and C(A0.6 + D0.2) groups, were treated with 0 (vehicle), 1, or 5 μM of either the SIRT6 inhibitor or rotenone, and cell viability was assessed after 48 h (Fig. 3E, F).

The AML group cells were relatively more sensitive to Ara-C and DNR than the R/R AML group cells (Fig. 3E, panels i–iii). However, these cells did not exhibit a significant response to SIRT6 inhibition. Under rotenone treatment, most of the AML group cells showed increased viability under the C(A0.6 + D0.061) and C(A0.6 + D0.2) conditions (Fig. 3F, panels i–ii) or no significant change (Fig. 3F, panel iii). Because one of the cytotoxic mechanisms of DNR involves the production of mitochondrial ROS and activation of intrinsic apoptosis, inhibition of mitochondrial function may impede this pathway, thereby partially reducing the death of cancer cells. The R/R AML group cells exhibited lower sensitivity to Ara-C and DNR than those in the AML group. In contrast, these cells exhibited a significant, dose-dependent decrease in viability in response to the SIRT6 inhibitor (Fig. 3E, panels iv–vi). Notably, two of these cells showed a dose-dependent decrease in viability following rotenone treatment (Fig. 3F, panels iv–v).

These findings, together with results for RHI-1 cells, indicated that the inhibition of DNA repair and OXPHOS enhanced the efficacy of Ara-C and DNR in the R/R AML group.

## Discussion

In addition to AML, most cancer cells predominantly metabolize carbohydrates and energy through glycolysis, followed by lactate fermentation, even in the presence of oxygen [36]. In contrast, aerobic respiration is predominant in cells derived from patients with R/R AML and in Ara-C-resistant cells [29, 37–39].

The findings of this study contribute to our understanding of the mechanisms of chemotherapy resistance in R/R AML, identifying mitochondrial metabolism, DNA repair, and ROS detoxification as the key pathways driving drug resistance. A comprehensive understanding of these pathways has significant clinical implications for the development of effective targeted therapies for patients with wild-type FLT3 R/R AML. Additionally, our results highlighted the critical role of mitochondrial metabolism in promoting chemotherapeutic resistance in Ara-C-resistant AML cells. Similarly, evidence from clinical studies suggests that disrupting mitochondrial pathways could be a potential strategy to improve treatment outcomes. For example, Pollyea et al. [40] demonstrated that the combination of venetoclax, a BCL-2 inhibitor, and azacitidine disrupted mitochondrial metabolism and effectively targeted leukemia stem cells, leading to better clinical outcomes in patients with AML. These findings highlight the therapeutic potential of targeting mitochondrial metabolism as a strategy to overcome resistance and improve the efficacy of existing chemotherapies such as Ara-C and DNR. Clinical trials on the effects of inhibiting mitochondrial metabolic pathways, especially OXPHOS complex I, could provide new therapeutic options for patients with resistant AML. Our study further highlights that RHI-1 cells rely on mitochondrial adaptations and enhanced DNA repair pathways to resist chemotherapy, confirming clinical evidence that combination therapies targeting these pathways can significantly improve patient outcomes. DiNardo et al. reported that combination treatment with venetoclax and hypomethylating agents, such as decitabine and azacytidine, improved the outcome of elderly patients with AML [41]. This combination exploits the metabolic weaknesses of leukemia cells, demonstrating the potential of integrated therapeutic approaches that inhibit mitochondrial metabolism. Additionally, our results revealed that ROS detoxification pathways play a significant role in chemotherapy resistance in RHI-1 cells, mirroring previous studies research that have shown the critical importance of ROS management in cancer cell survival. Herst and Berridge [42] showed that enhanced ROS detoxification affects cellular oxygen consumption and contributes to chemoresistance. Therefore, addressing ROS detoxification could represent a valuable approach to reduce resistance and enhance the efficacy of chemotherapy in patients with AML. Overall, these clinical implications underscore the need for further research on metabolic adaptations in leukemia cells, including mitochondrial metabolism and ROS detoxification, to develop effective chemotherapeutic regimens. Additionally, strategically targeting these resistance pathways may improve patient outcomes, particularly in patients with limited treatment options.

Conclusively, our study provides valuable insights into the mechanisms underlying chemotherapy failure in FLT3-ITD wild-type R/R AML. Disrupting mitochondrial metabolic proteins in FLT3-ITD wild-type R/R AML cells can improve the efficacy of the Ara-C and DNR regimens.

## Materials and methods

### Cell cultures and cell viability test

The SHI-1 (CVCL_2191), an acute myeloid leukemia cell line, was obtained from the Deutsche Sammlung von Mikroorganismen und Zellkulturen (DSMZ, Braunschweig, Germany). DSMZ confirmed the authentication of the SHI-1 cell line. Cytarabine (Ara-C) resistant SHI-1 cells (RHI-1) were provided as a kind gift by Molecular Medicine Finland, University of Helsinki. Cells were maintained in RPMI 1640 medium (Catalog No. LM-011-03; Welgene, Seoul, Korea) supplemented with L-glutamine (2 mM; Catalog No. 25030-081; Thermo Fisher Scientific, Waltham, MA, USA), 10% fetal bovine serum (FBS; Catalog No. F2442; Sigma-Aldrich, St. Louis, MO, USA), and 1% antibiotic-antimycotic solution (100×; Catalog No. 15240-062; Thermo Fisher Scientific). Patient samples were collected from six individuals with AML, divided into two groups: three treatment-responsive cases (AML-P1, AML-P2, AML-P3) and three relapsed/refractory (R/R) cases (R/R-P1, R/R-P2, R/R-P3). Clinical details are provided in Table S5. All samples were obtained in accordance with the ethical guidelines prescribed in the Declaration of Helsinki. The study protocol was approved by the Institutional Review Board of Korea University Anam Hospital (IRB No. 2020AN0415), and informed consent was obtained from all participants before sample collection. Cells from patient samples were counted and resuspended in MCM (MCM; Catalog No. C-28030; PromoCell) with 0.5 μg/mL gentamicin (50 mg/mL; Catalog No. 15750-060; Thermo Fisher Scientific) and 2.5 μg/mL amphotericin (250 µg/mL; Catalog No. 15290-018; Thermo Fisher Scientific). Cells were cultured at 37 °C in a humidified atmosphere containing 5% CO_2_ and were passaged every two to three days. For cell viability assays, SHI-1 and RHI-1 cells were seeded at a density of 15,000 cells per 100 μL of culture medium into each well of a 96-well microplate (Corning Costar, Catalog No. 3595) and incubated for 24 h. Following incubation, cells were treated with drugs either as single doses or in combination, as depicted in Figs 1, 3. Cell viability was assessed at 0, 24, and 48 h post-treatment using the Cell Counting Kit-8 (CCK-8; Dojindo Molecular Technologies, Inc., Kumamoto, Japan). Absorbance was measured at 450 nm using a microplate reader (e.g., BioTek Synergy HT). Each treatment condition was performed in triplicate wells, and the entire assay was independently repeated three times to ensure reproducibility [43].

### FLT3-ITD mutation fragment analysis

DNA from SHI-1 cells and RHI-1 cells was harvested using DNA extraction kit (TaKaRa MiniBEST Universal Genomic DNA Extraction Kit Ver.5.0; Takara Bio). FLT3-ITD mutations were analyzed in extracted DNA by fragment analysis through PCR. PCR was performed for both the DNA of SHI-1 cells and the DNA of RHI-1 cells using specific primers (FLT3 forward primer: 5′-FAM-AGCAATTTAGGTATGAAAGCCAGCTA-3′ and reverse primer-5′-CTTTCAGCATTTTGACGGCAACC-3′. Beta474 forward primer-5′-PET-CCAGAAGAGCCAAGGACAGGTACG-3′ and reverse primer-5′- AGATCCCCAAAGGACTCAAAGAACC-3′) under the following conditions: 1 cycle at 95 °C for 5 min, 35 cycles at 95 °C for 20 s, 58 °C for 40 s, 72 °C for 30 s, and 1 cycle for 72 °C for 5 min using a Veriti™ 96-Well Fast Thermal Cycler (Thermo Fisher, Waltham, MA, USA). Capillary electrophoresis and Fragment analysis were performed in an 3500 Genetic Analyzer (Thermo Fisher, Waltham, MA, USA).

### Quantitative analysis of Ara-C and DNR uptake

SHI-1 cells and RHI-1 cells were seeded in cell culture flasks (SPL Life Science, Seoul, Korea) and then treated with 0.64 μM Ara-C alone, 0.61 or 2 μM DNR alone, or DNR combined with 0.64 μM Ara-C, respectively. After 1, 24, and 48 h, live and dead cells were separated using Histopaque-10771 (Sigma-Aldrich, Catalog No. H10771), and each harvested cells was washed with PBS (PBS; Thermo Fisher Scientific, Catalog No. 10010023). Cells were resuspended with water, lysed by freeze-thaw method (alternating between −80 °C and room temperature), and centrifuged at 14,000 × *g*, 4 °C for 10 min. The supernatants were taken, and ACN (ACN; J.T. Baker, Catalog No. 270942) was added at a 1:1 ratio to precipitate proteins, and precipitated proteins were removed by centrifugation at 7500 × *g*, 4 °C for 5 min. The protein-free supernatants were desalted by Oasis HLB cartridge (Waters Corporation, Catalog No. WAT053933), and the buffer was removed using Concentrator Plus (Eppendorf, Catalog No. 5632). Samples were reconstituted with mobile phase (0.1% formic acid in water), and the amount of cellular-uptaken cytarabine and daunorubicin was quantitated. Quantitative analysis was performed using TQMS with LCMS-8050 spectrometer (Shimadzu, Kyoto, Japan) in both positive and negative ion modes. The source parameters were 200 (interface), 250 (DL), and 200 °C (heating gas temperatures). The flow rates of nebulizing, heating, and drying gases were 3, 10, and 10 L/min, respectively. ACQUITY BEH C18 1.7 μm VANGUARD Pre-co (Waters, Catalog No. 186003890) and Shimadzu-pack C18 3 μm (Shimadzu, Catalog No. 80348-8054) were used for L.C. separation. The temperature setting of L.C. was 40 °C, and the injection volume of the sample was 10 μL. Water containing 0.1% formic acid (A) and ACN containing 0.1% formic acid (B) were used as solvents, and the flow rate was 0.1 mL/min. L.C. time program was scheduled as follows: 0–5 min, 0% B; 5–50 min, the concentration of B increased 0–80%; 50–60 min, sharp reduction to 0% B. *m/z* = 244.10 represented the protonated cytarabine ion that fragmented to *m/z* = 112.05, 95.00, and 69.05. *m/z* = 528.80 represented the protonated daunorubicin ion that fragmented to *m/z* = 322.10, 364.10, and 307.00.

The amount of Ara-C in A0.64, C(A0.64 + D0.61), and C(A0.64 + D2)-treated live cells was measured at 1, 24, and 48 h after treatment. Similarly, we quantified DNR in D0.61, D2, C(A0.64 + D0.61), and C(A0.64+D2)-treated cells at the same time points. [Drug] cells were calculated by dividing the drug amounts by the number of cells used for lysis.

### Cell lysis and peptide digestion

Cells were washed once with PBS and lysed for 30 min in ice-cold lysis urea buffer 8 M urea; 75 mM NaCl, 50 mM Tris HCl pH 8.0, 1 mM EDTA, 2 μg/mL aprotinin (Sigma, A6103), 10 μg/mL leupeptin (Roche, #11017101001), 1 mM PMSF (Sigma, 78830), 10 mM NaF, 5 mM sodium butyrate, 5 mM iodoacetamide (Sigma, A3221), Phosphatase Inhibitor Cocktail 2 (1:100, Sigma, P5726), Phosphatase Inhibitor Cocktail 3 (1:100, Sigma, P0044). Lysates were centrifuged at 20,000 × *g* for 10 min, and protein concentrations of the clarified lysates were measured via BCA assay. After BCA assay, lysates were reduced for 60 min with 5 mM dithiothreitol (Thermo Scientific, 20291) and alkylated for 60 min with 10 mM iodoacetamide. Samples were then diluted 1:4(v/v) with 50 mM Tris HCl, pH 8.0, to reduce the urea concentration to 2 M. Finally lysates were digested overnight at 37 °C with trypsin in a 1:30 enzyme-to-substrate ratio on a shaker. After digestion, Samples were acidified to quench the trypsin activation (v/v = 1%) and centrifuged at 2000 × *g* for 5 min. Peptide mixtures were desalted on tC18 SepPak columns (Waters, 500 mg WAT036790). Columns were conditioned with 2 × 5 ml 100% acetonitrile and 2 × 5 ml 50% acetonitrile/0.1% formic acid washes, and equilibrated with 4 × 5 ml 0.1% trifluoroacetic acid. After loading the sample onto the column, samples were desalted with 3 × 5 ml 0.1% trifluoroacetic acid washes and 1 × 5 ml 1% formic acid wash. Peptides were eluted with 2 × 3 ml 50% acetonitrile/0.1% formic acid. Eluted peptide samples were placed in a vacuum evaporator to evaporate the solvent. After evaporation, 18 samples peptide concentrations were measured via BCA assay.

### Overall method for Quantitative proteomic/phosphoproteomic profiling

For these analyses, SHI-1 and RHI-1 cells in the control (untreated), D0.61, D2, C(A0.64 + D0.61), and C(A0.64 + D2) groups were harvested after 48 h (*n* = 3 samples/group, making a total of 18 samples) to obtain cell lysates. Cell lysates were digested with trypsin, followed by 18 plex-tandem mass tag labeling (18 plex-TMTpro) (Fig. S2A). All individually TMT-labeled samples were pooled into one tube, and 5% of the pooled TMT-labeled proteome was used for global proteome profiling, whereas 95% of the pooled proteome was used for phosphoproteome profiling via specific enrichment of phosphopeptides using ferric nitrilotriacetate immobilized metal affinity chromatography [44]. Both the TMT-labeled proteome and the enriched phosphoproteome were subjected to concatenated fractionation into 30 fractions using an offline mid-pH reversed-phase fractionation system. All fractionated samples were analyzed via Orbitrap Eclipse using the synchronous precursor selection-MS3 (SPS-MS3) method, resulting in the profiling of 5816 quantifiable global proteins and 2557 phosphoproteins.

### TMTpro 18plex labeling and IMAC

Total 5.4 mg of 18 samples, corresponding to 0.3 mg from each sample, were labeled with 0.5 mg TMTpro reagents in 20% ACN, 50 mM HEPES for 2 h. TMT 126/127 N/128 N, 131 N/129 N/130 N, 130 C/131 C/132 C, 132 N/133 N/134 C, 127 C/128 C/129 C, and 135 N/133 C/134 N were utilised to peptides labeling from drug-untreated SHI-1, drug-untreated RHI-1, D0.61-, D2-, C(A0.64+D0.61)-, and C(A0.64+D2)-treated RHI-1 cells, respectively. The TMT labeling reaction was quenched by adding 4 µL of 5% hydroxylamine for 15 min at room temperature. Samples were combined and placed in a vacuum evaporator to evaporate the solvent. Phopshopeptides were enriched by using Fe-NTA phosphopeptide enrichment kit (Thermo Fisher Scientific, A32992). Eluents were combined, and combined sample was placed in a vacuum evaporator to evaporate the solvent. Peptide concentrations were measured via BCA assay.

### High-pH off-line fraction

To increase the analytical depth, offline mid-pH reversed-phase fractionation was performed. Combined 54 μg of peptide sample was separated using the nanoACQUITY UPLC system (Waters) with an in-house-packed column (320 μm i.d. x 55 cm) using 3 μm Jupyter C18 particles (Phenomenex). The LC flow rate was 7 μl/min with a 92 min linear gradient ranging from 98% solvent A (10 mM ABC, pH 8) to 40% solvent B (90% acetonitrile with 10 mM ABC). Each fraction was collected every 1 min and automatically concatenated into final 30 pools using TriVersa NanoMate robot (Advion). All the fractions were dried and resuspended in 10 μl of 25 mM ABC for LC-MS3 analysis.

### LC-MS3 analysis

Synchronous precursor selection-MS3 analysis was carried out for each fraction using Orbitrap Eclipse Tribrid MS (Thermo Fisher Scientific) coupled with a NanoAcquity UPLC equipped with an in-house packed trap (150 μm i.d. x 3 cm) and analytical column (75 μm i.d. x 100 cm) using 3 μm Jupiter C18 particles. A linear gradient of solvent A (0.1% formic acid) and solvent B (100% ACN, 0.1% formic acid) was applied at a flow rate 300 nl/min as follows: 0–5 min, 2–5% solvent B; 5–15 min, 5–10% solvent B; 15–190 min, 10–30% solvent B; 190–195 min, 30–80% solvent B; 195–200 min 80% solvent B isocratic, followed by 35 min re-equilibration of the column. Full MS scans (m/z 375-1575) were acquired at a resolution of 120k (at m/z 200). Higher-energy collisional dissociation (HCD) fragmentation of precursor ions with charge 2–7 was performed under 30% of normalized collision energy (NCE) via precursor isolation within 0.7 Th window. Dynamic exclusion value was set to 60 s. The MS2 scans were acquired at a resolution of 15k (maximum precursor ion injection time (ITmax) of 60 ms and automatic gain control (AGC) of 5E4). The 10 most intense MS2 fragment ions were first isolated at 0.4 Th of precursor isolation width and then synchronously isolated for HCD MS3 (ITmax of 54 ms, AGC of 2.5E5, and NCE of 65%) at 3 Th of isolation width. Raw data were analyzed with Proteome Discoverer 2.2 (Thermo Fisher Scientific; RRID:SCR_014477) with the TMTpro reporter ion quantification workflows using the standard (Thermo Fisher Scientific (RRID:SCR_008452)). Spectra were searched using the Sequest search engine using the homo sapiens database obtained from Uniprot with trypsin set as enzyme (RRID:SCR_002380). Carbamidomethylation of cysteine residues (+57.021 Da), TMT of peptide N termini and lysine residues (+229.163 Da) were set as static modifications, while the oxidation of methionine residues (+15.995 Da) was set as a variable modification. Peptide-spectrum matches (PSMs) were adjusted to a 1% false discovery rate (FDR). To assign DE proteins, all channels’ intensities were normalized, and log2FC and corrected p-value via the Benjamini–Hochberg were used for further analysis.

### Identification of DEPs and differentially expressed phosphopeptides

We computed the log_2_-fold-change in TMT intensity between drug-untreated or treated with D0.61, D2, C(A0.64+D0.61), or C(A0.64+D2) RHI-1 cells and drug-untreated SHI-1 cells. Then, we identified the differentially expressed proteins (DEPs) with p-value<0.05 and absolute log_2_-fold-changes > 1 (2-fold) (Table 2). The differentially expressed phospho-proteins (phos-DEPs) were identified in the same way with the DEPs.

**Table 2:** Table of defined terms for fold-change calculation.

	Treatment					Term
Fold-change	untreated	D0.61	D2	C(A0.64 + D0.61)	C(A0.64 + D2)	
${\bf{l}}{{\bf{og}}}_{{\boldsymbol{2}}}\left(\frac{{\boldsymbol{TMT\; inten}}{\boldsymbol{.}}}{{\boldsymbol{TMT\; inten}}{\boldsymbol{.}}}\right)$	RHI-1					RHI-1(untreated)
	SHI-1					
${\bf{l}}{{\bf{og}}}_{{\boldsymbol{2}}}\left(\frac{{\boldsymbol{TMT\; intens}}{\boldsymbol{.}}}{{\boldsymbol{TMT\; inten}}{\boldsymbol{.}}}\right)$		RHI-1				RHI-1(0.61 DNR)
	SHI-1					
${\bf{l}}{{\bf{og}}}_{{\boldsymbol{2}}}\left(\frac{{\boldsymbol{TMT\; inten}}{\boldsymbol{.}}}{{\boldsymbol{TMT\; inten}}{\boldsymbol{.}}}\right)$			RHI-1			RHI-1(2 DNR)
	SHI-1					
${\bf{l}}{{\bf{og}}}_{{\boldsymbol{2}}}\left(\frac{{\boldsymbol{TMT\; inten}}{\boldsymbol{.}}}{{\boldsymbol{TMT\; inten}}{\boldsymbol{.}}}\right)$				RHI-1		RHI-1(0.61 Comb)
	SHI-1					
${\bf{l}}{{\bf{og}}}_{{\boldsymbol{2}}}\left(\frac{{\boldsymbol{TMT\; inten}}{\boldsymbol{.}}}{{\boldsymbol{TMT\; inten}}{\boldsymbol{.}}}\right)$					RHI-1	RHI-1(2 Comb)
	SHI-1					

### Protein extraction and immunoblotting

Cell lysates were prepared by lysing cells in RIPA buffer containing phosphatase and protease inhibitors. Bradford and bicinchoninic acid protein assay kits (Thermo Fisher Scientific) were used to determine the protein concentration in whole lysates. A total of 20 μg of each cell lysate was loaded on 12% gels for SDS-PAGE and transferred to a polyvinylidene fluoride membrane (Bio-Rad Laboratories, Inc., California, USA). The following antibodies were used at a dilution of 1:1000, rabbit anti-Sirtuin 6 (SIRT6) antibody (cell signaling technology, Beverly, MA, USA; RRID:AB_2188926), rabbit anti-catalase (CAT) antibody (cell signaling technology, Beverly, MA, USA; RRID:AB_2798079), rabbit anti-phosphoDNA-dependent protein kinase (phos-DNA-PK) antibody (cell signaling technology, Beverly, MA, USA; RRID:AB_2939025), and β-actin (Invitrogen). After probing with the appropriate species-specific horseradish peroxidase (HRP)-linked secondary antibodies (1:10000), the proteins were detected using EZ-Western and EZ-Western Lumi Femto kits (DoGenBio, Seoul, Korea) and a LAS mini 4000 (GE Healthcare, Amersham, UK). Data were analyzed using the ImageJ software (NIH, Bethesda, MD, USA; RRID:SCR_003070).

### SIRT6 knockdown using siRNA transfection

SIRT6 knockdown was performed using AccuTarget™ Genome-wide Predesigned siRNA (BioRP, 100 nmole, siRNA No: 51548-1, Human SIRT6; Bioneer, Daejeon, Korea) and Lipofectamine™ RNAiMAX Transfection Reagent (Catalog No. 13778150; Invitrogen, Thermo Fisher Scientific, Waltham, MA, USA) according to the manufacturer’s protocol.

### Statistical analysis

We assessed the homogeneity of variances among the groups using an F-test, and evaluated the normality of the data by generating Q-Q plots. The results confirmed that the variances across groups were similar and that the data were approximately normally distributed. Trend analysis was performed using simple linear regression, and the resulting trend *p* value is reported. For comparisons between two groups, p-values were determined using an unpaired *t*-test (two-tailed). Center values are defined as the mean, and error bars represent the standard error of the mean (SEM). All details regarding the number of cells, experimental replicates, and significance level are described in each method section and the figure captions. Significance of comparisons is indicated in figures and supplemental data as ∗*p* < 0.05, ∗∗*p* < 0.01, ∗∗∗*p* < 0.001, ∗∗∗∗*p* < 0.0001.

## Supplementary information

Supplementary Materials

Original Western blot

Supplementary Data

## Data Availability

All data generated or analyzed during this study are included in this published article and its supplementary information files. The mass spectrometry proteomics data generated during this study are available at the ProteomeXchange Consortium via the PRIDE partner repository, with the dataset identifier [PXD054797].

## References

[1] Döhner H, Weisdorf DJ, Bloomfield CD. Acute myeloid leukemia. N Engl J Med. 2015;373:1136–52. 26376137 10.1056/NEJMra1406184

[2] Lowenberg B, Downing JR, Burnett A. Acute myeloid leukemia. New Engl J Med. 1999;341:1051–62. 10502596 10.1056/NEJM199909303411407

[3] Ishii H, Yano S. New therapeutic strategies for adult acute myeloid leukemia. Cancers. 2022;14:2806. 35681786 10.3390/cancers14112806 PMC9179253

[4] Milligan DW, Wheatley K, Littlewood T, Craig JIO, Burnett AK. Group ftNHOCS. Fludarabine and cytosine are less effective than standard ADE chemotherapy in high-risk acute myeloid leukemia, and addition of G-CSF and ATRA are not beneficial: results of the MRC AML-HR randomized trial. Blood. 2006;107:4614–22. 16484584 10.1182/blood-2005-10-4202

[5] Morris TA, DeCastro CM, Diehl LF, Gockerman JP, Lagoo AS, Li Z, et al. Re-induction therapy decisions based on day 14 bone marrow biopsy in acute myeloid leukemia. Leuk Res. 2013;37:28–31. 23046833 10.1016/j.leukres.2012.09.016 PMC3753071

[6] Network TCGAR. Genomic and epigenomic landscapes of adult de novo acute myeloid leukemia. New Engl J Med. 2013;368:2059–74. 23634996 10.1056/NEJMoa1301689 PMC3767041

[7] Tsuchiya S, Yamabe M, Yamaguchi Y, Kobayashi Y, Konno T, Tada K. Establishment and characterization of a human acute monocytic leukemia cell line (THP-1). Int J Cancer. 1980;26:171–6. 6970727 10.1002/ijc.2910260208

[8] Malani D, Murumagi A, Yadav B, Kontro M, Eldfors S, Kumar A, et al. Enhanced sensitivity to glucocorticoids in cytarabine-resistant AML. Leukemia. 2017;31:1187–95. 27833094 10.1038/leu.2016.314 PMC5420795

[9] Huang DW, Sherman BT, Lempicki RA. Bioinformatics enrichment tools: paths toward the comprehensive functional analysis of large gene lists. Nucleic Acids Res. 2008;37:1–13. 19033363 10.1093/nar/gkn923 PMC2615629

[10] Kanehisa M, Goto S. KEGG: Kyoto encyclopedia of genes and genomes. Nucleic Acids Res. 2000;28:27–30. 10592173 10.1093/nar/28.1.27 PMC102409

[11] Siddiqui A, Ceppi P. A non-proliferative role of pyrimidine metabolism in cancer. Mol Metab. 2020;35:100962. 32244187 10.1016/j.molmet.2020.02.005 PMC7096759

[12] Boukalova S, Hubackova S, Milosevic M, Ezrova Z, Neuzil J, Rohlena J. Dihydroorotate dehydrogenase in oxidative phosphorylation and cancer. Biochimica Et Biophys Acta BBA Mol Basis Dis. 2020;1866:165759. 10.1016/j.bbadis.2020.165759 32151633

[13] Huang R, Chen H, Liang J, Li Y, Yang J, Luo C, et al. Dual role of reactive oxygen species and their application in cancer therapy. J Cancer. 2021;12:5543–61. 34405016 10.7150/jca.54699 PMC8364652

[14] Trachootham D, Alexandre J, Huang P. Targeting cancer cells by ROS-mediated mechanisms: a radical therapeutic approach?. Nat Rev Drug Discov. 2009;8:579–91. 19478820 10.1038/nrd2803

[15] Yang F, Teves SS, Kemp CJ. Henikoff S. Doxorubicin, DNA torsion, and chromatin dynamics. Biochim Et Biophys Acta BBA Rev Cancer. 2014;1845:84–9. 10.1016/j.bbcan.2013.12.002 PMC3927826 24361676

[16] Christidi E, Brunham LR. Regulated cell death pathways in doxorubicin-induced cardiotoxicity. Cell Death Dis. 2021;12:339. 33795647 10.1038/s41419-021-03614-x PMC8017015

[17] Zhao J, Zhang N, Ma X, Li M, Feng H. The dual role of ferroptosis in anthracycline-based chemotherapy includes reducing resistance and increasing toxicity. Cell Death Discov. 2023;9:184. 37344500 10.1038/s41420-023-01483-1 PMC10284859

[18] Cao X, Fang L, Gibbs S, Huang Y, Dai Z, Wen P, et al. Glucose uptake inhibitor sensitizes cancer cells to daunorubicin and overcomes drug resistance in hypoxia. Cancer Chemother Pharmacol. 2007;59:495–505. 16906425 10.1007/s00280-006-0291-9

[19] Rai Y, Yadav P, Kumari N, Kalra N, Bhatt AN. Hexokinase II inhibition by 3-bromopyruvate sensitizes myeloid leukemic cells K-562 to anti-leukemic drug, daunorubicin. Bioscience Rep. 2019;39:BSR20190880. 10.1042/BSR20190880 PMC6757186 31506393

[20] Liu Y, Zhou Q, Song S, Tang S. Integrating metabolic reprogramming and metabolic imaging to predict breast cancer therapeutic responses. Trends Endocrinol Metab. 2021;32:762–75. 34340886 10.1016/j.tem.2021.07.001

[21] Xie Y, Hou W, Song X, Yu Y, Huang J, Sun X, et al. Ferroptosis: process and function. Cell Death Differ. 2016;23:369–79. 26794443 10.1038/cdd.2015.158 PMC5072448

[22] Bean JF, Qiu Y-Y, Yu S, Clark S, Chu F, Madonna MB. Glycolysis inhibition and its effect in doxorubicin resistance in neuroblastoma. J Pediatr Surg. 2014;49:981–4. 24888847 10.1016/j.jpedsurg.2014.01.037

[23] Klinakis A, Karagiannis D, Rampias T, Targeting DNA. repair in cancer: current state and novel approaches. Cellular Mol Life Sci. 2020;77:677–703. 31612241 10.1007/s00018-019-03299-8 PMC11105035

[24] Kciuk M, Bukowski K, Marciniak B, Kontek R. Advances in DNA repair—emerging players in the arena of eukaryotic DNA repair. Int J Mol Sci. 2020;21:3934. 32486270 10.3390/ijms21113934 PMC7313471

[25] Chen P-H, Wu J, Ding C-KC, Lin C-C, Pan S, Bossa N, et al. Kinome screen of ferroptosis reveals a novel role of ATM in regulating iron metabolism. Cell Death Differ. 2020;27:1008–22. 31320750 10.1038/s41418-019-0393-7 PMC7206124

[26] Davison K, Côté S, Mader S, Miller WH. Glutathione depletion overcomes resistance to arsenic trioxide in arsenic-resistant cell lines. Leukemia. 2003;17:931–40. 12750708 10.1038/sj.leu.2402876

[27] Nandi A, Yan L-J, Jana CK, Das N. Role of catalase in oxidative stress- and age-associated degenerative diseases. Oxidative Med Cell Longev. 2019;2019:9613090. 10.1155/2019/9613090 PMC6885225 31827713

[28] Selheim F, Aasebø E, Bruserud Ø, Hernandez-Valladares M. High mitochondrial protein expression as a potential predictor of relapse risk in acute myeloid leukemia patients with the monocytic FAB subtypes M4 and M5. Cancers. 2024;16:8. 10.3390/cancers16010008 PMC10778527 38201437

[29] Stratmann S, Vesterlund M, Umer HM, Eshtad S, Skaftason A, Herlin MK, et al. Proteogenomic analysis of acute myeloid leukemia associates relapsed disease with reprogrammed energy metabolism both in adults and children. Leukemia. 2023;37:550–9. 36572751 10.1038/s41375-022-01796-7 PMC9991901

[30] Murray HC, Enjeti AK, Kahl RGS, Flanagan HM, Sillar J, Skerrett-Byrne DA, et al. Quantitative phosphoproteomics uncovers synergy between DNA-PK and FLT3 inhibitors in acute myeloid leukaemia. Leukemia. 2021;35:1782–7. 33067575 10.1038/s41375-020-01050-y PMC8179851

[31] Chalkiadaki A, Guarente L. The multifaceted functions of sirtuins in cancer. Nat Rev Cancer. 2015;15:608–24. 26383140 10.1038/nrc3985

[32] Fiorentino F, Mai A, Rotili D. Emerging therapeutic potential of SIRT6 modulators. J Med Chem. 2021;64:9732–58. 34213345 10.1021/acs.jmedchem.1c00601 PMC8389836

[33] Becherini P, Caffa I, Piacente F, Damonte P, Vellone VG, Passalacqua M, et al. SIRT6 enhances oxidative phosphorylation in breast cancer and promotes mammary tumorigenesis in mice. Cancer Metab. 2021;9:6. 33482921 10.1186/s40170-021-00240-1 PMC7821730

[34] Klein MA, Denu JM. Biological and catalytic functions of sirtuin 6 as targets for small-molecule modulators. J Biol Chem. 2020;295:11021–41. 32518153 10.1074/jbc.REV120.011438 PMC7415977

[35] Heinz S, Freyberger A, Lawrenz B, Schladt L, Schmuck G, Ellinger-Ziegelbauer H. Mechanistic investigations of the mitochondrial complex i inhibitor rotenone in the context of pharmacological and safety evaluation. Sci Rep. 2017;7:45465. 28374803 10.1038/srep45465 PMC5379642

[36] Warburg O, Wind F, Negelein E. The metabolism of tumors in the body. J Gen Physiol. 1927;8:519–30. 19872213 10.1085/jgp.8.6.519 PMC2140820

[37] Forte D, García-Fernández M, Sánchez-Aguilera A, Stavropoulou V, Fielding C, Martín-Pérez D, et al. Bone marrow mesenchymal stem cells support acute myeloid leukemia bioenergetics and enhance antioxidant defense and escape from chemotherapy. Cell Metab. 2020;32:829–43.e9. 32966766 10.1016/j.cmet.2020.09.001 PMC7658808

[38] de Beauchamp L, Himonas E, Helgason GV. Mitochondrial metabolism as a potential therapeutic target in myeloid leukaemia. Leukemia. 2022;36:1–12. 34561557 10.1038/s41375-021-01416-w PMC8727299

[39] Sharon D, Cathelin S, Mirali S, Di Trani JM, Yanofsky DJ, Keon KA, et al. Inhibition of mitochondrial translation overcomes venetoclax resistance in AML through activation of the integrated stress response. Sci Transl Med. 2019;11:eaax2863. 31666400 10.1126/scitranslmed.aax2863

[40] Pollyea DA, Stevens BM, Jones CL, Winters A, Pei S, Minhajuddin M, et al. Venetoclax with azacitidine disrupts energy metabolism and targets leukemia stem cells in patients with acute myeloid leukemia. Nat Med. 2018;24:1859–66. 30420752 10.1038/s41591-018-0233-1 PMC7001730

[41] DiNardo CD, Pratz K, Pullarkat V, Jonas BA, Arellano M, Becker PS, et al. Venetoclax combined with decitabine or azacitidine in treatment-naive, elderly patients with acute myeloid leukemia. Blood. 2019;133:7–17. 30361262 10.1182/blood-2018-08-868752 PMC6318429

[42] Herst PM, Berridge MV. Cell surface oxygen consumption: a major contributor to cellular oxygen consumption in glycolytic cancer cell lines. Biochimica et Biophysica Acta (BBA). Bioenergetics. 2007;1767:170–7. 10.1016/j.bbabio.2006.11.018 17266920

[43] Tominaga H, Ishiyama M, Ohseto F, Sasamoto K, Hamamoto T, Suzuki K, et al. A water-soluble tetrazolium salt useful for colorimetric cell viability assay. Anal Commun. 1999;36:47–50.

[44] Mertins P, Tang LC, Krug K, Clark DJ, Gritsenko MA, Chen L, et al. Reproducible workflow for multiplexed deep-scale proteome and phosphoproteome analysis of tumor tissues by liquid chromatography–mass spectrometry. Nat Protoc. 2018;13:1632–61. 29988108 10.1038/s41596-018-0006-9 PMC6211289

