# Supplementary material for: Mechanisms of chemotherapy failure in refractory/relapsed acute myeloid leukemia: the role of cytarabine resistance and mitochondrial metabolism
Source: Cell Death Dis. 2025 Apr 23;16(1):331. doi: 10.1038/s41419-025-07653-6 (PMC12019594; doi:10.1038/s41419-025-07653-6)
Supplement: Supplementary file 1 — Supplementary Materials [file 41419_2025_7653_MOESM1_ESM.docx]

Supplementary Material

Mechanisms of chemotherapy failure in refractory/relapsed acute myeloid leukemia: The role of cytarabine resistance and mitochondrial metabolism

Soo Yeon Chae^1,2,‡^, Se-Young Jang^3,‡^, Jinhui Kim^1^, Sehyun Hwang^1,2^, Disha Malani^6,7^, Olli Kallioniemi^7,8^, Seung Gyu Yun^5,*^, Jong-Seo Kim^3,4,*^, Hugh I. Kim^1,2,9,*^

^1^ Department of Chemistry, Korea University, Seoul, 02841, Republic of Korea

^2^ Center for Proteogenome Research, Korea University, Seoul, 02841, Republic of Korea

^3^ School of Biological Sciences, Seoul National University, Seoul, 08826, Korea

^4^ Center for RNA Research, Institute of Basic Science, Seoul National University, Seoul, 08826, Korea

^5^ Department of Laboratory Medicine, Korea University College of Medicine, Seoul, Korea.

^6^ Department of Medical Oncology, Dana-Farber Cancer Institute, Boston, Massachusetts.

^7^ Institute for Molecular Medicine Finland, FIMM, University of Helsinki, Helsinki, Finland

^8^ Science for Life Laboratory, Department of Oncology and Pathology, Karolinska Institutet, Solna, Sweden

^9^ Lead Contact

‡ These authors contributed equally.

*Correspondence: [koryun@korea.ac.kr](mailto:koryun@korea.ac.kr), [jongseokim@snu.ac.kr](mailto:jongseokim@snu.ac.kr), [hughkim@korea.ac.kr](mailto:hughkim@korea.ac.kr)

**Table of Contents**

| **Contents** | | | |  |
| --- | --- | --- | --- | --- |
| **Supplementary Discussion** | | | |  |
| **Supplemental Data Items** | | | |  |
| **Supplementary Table 1.** | | The viability of SHI-1 cells at 48h decreased with increasing concentration of cytarabine. | |  |
| **Supplementary Table 2.** | | The viability of RHI-1 cells at 48h decreased with increasing concentration of cytarabine. | |  |
| **Supplementary Table 3** | | [Drug]_cell_ in SHI-1The viability of RHI-1 cells at 48h | |  |
| **Supplementary Table 4** | | log2(Fold-change) of Ara-C resistance-related genes | |  |
| **Supplementary Table 5** | | **C**linical characteristics of AML patients | |  |
| **Supplementary Figure 1** | | FLT3-ITD Mutation Analysis and Cell Viability Responses to Ara-C and DNR Combinations in SHI-1 and RHI-1 Cell Lines | |  |
| **Supplementary Figure 2** | | Global proteome and phosphoproteome MS3 profiling of AML cells treated with DNR or Ara. | |  |
| **Supplementary Figure 3** | | Sample correlation analysis | |  |
| **Supplementary Figure 4** | | Gene ontology analysis and cluster network | |  |
| **Supplementary Figure 5** | | Metabolic Remodeling in SHI-1 and RHI-1 Cells Under Ara-C and DNR Treatment | |  |
| **Supplementary Figure 6** | | Original western blots used in Figure 2. | |  |
|  | | |  |  |

**Supplementary Discussion**

**1. Drug tolerance of Ara-C resistant AML cells**

Ara-C and DNR treatments significantly decreased the viability of SHI-1 cells in a concentration-dependent manner within 48 h. Specifically, the viabilities of SHI-1 cells in the A0.16 to A0.64 treatment groups were 73 to 55% (Fig. 1A, B). Additionally, the viabilities of cells in the D0.08 to D2 groups were decreased 60 to 11%. The cell viability results for the combination treatments followed a similar pattern to that of the single treatments with DNR. Overall, these results indicate that treatments with Ara-C or DNR effectively decreased SHI-1 cell viability. Although the combination treatment decreased cell viability to the same extent as DNR monotherapy, no additional synergistic effects were detected.

Compared to SHI-1 cells, RHI-1 cells exhibited resistance to Ara-C and DNR (*p* < 0.01) treatments (Fig. 1A and B). Specifically, the viabilities of RH1-1 cells in the A0.16, A0.32, and A0.64 groups were 100, 101, and 101%, respectively. Although DNR treatment was cytotoxic to RHI-1 cells, its effect was lower than that in SHI-1 cells. The viabilities of RHI-1 cells in the D0.08 (78%), D0.16 (72%), D0.2 (71%), D0.3 (60%), D0.61 (46%), and D2 (14%) groups were 1.3–4 times higher than those of SHI-1 cells under the same treatments. Additionally, the viabilities of RHI-1 cells in the C(A0–A0.64+D0.61) and C(A0–A0.64+D2) groups ranged from 46–47% and 14–15%. Despite the different mechanisms of action of DNR and Ara-C, RHI-1 cells exhibited significant tolerance to DNR, regardless of the presence or absence of Ara-C. Collectively, RHI-1 cells were resistant to both Ara-C and DNR and the combined treatments.

**2. Intracellular drug concentrations in SHI-1/RHI-1 cells**

The [Ara-C]_cell_ values of SHI-1 cells in the A0.64 groups were increased 0.35–0.38 nmol/10^6^ cells up to 48 h (Fig. 1C-left, Table S3), whereas those of cells in the C(A0.64+D0.61) groups were increased 0.28–0.37 nmol/10^6^ cells within 48 h (Fig. 1C, middle). Additionally, the [Ara-C]_cell_ values of SHI-1 cells in the C(A0.64+D2) groups were increased 0.22 to 0.37 nmol/10^6^ cells within 48 h (Fig. 1C, right panel). The [Ara-C]_cell_ values of RHI-1 cells in the A0.64 group were 0.27, 0.27, and 0.29 nmol/10^6^ cells at 1, 24, and 48 h, respectively (Fig. 1F, left), whereas those of cells in the C(A0.64+D0.61) group were 0.33, 0.40, and 0.44 nmol/10^6^ cells, respectively (Fig. 1F, middle). Furthermore, the [Ara-C]_cell_ values of RHI-1 cells in the C(A0.64+D2) group were 0.34, 0.50, and 0.52 nmol/10^6^ cells at 1, 24, and 48 h, respectively (Fig. 1F, right).

Ara-C uptake by SHI-1 cells was lower under combined treatments, especially in the C(A0.64+D0.61) group, than under single treatments at 1 h and 24 h but increased at 48 h, suggesting that the early uptake of Ara-C was hindered by DNR. Consistent with the initial intracellular drug concentration, the viability of SHI-1 cells in the C(A0–0.64+D0.61 and 2) group showed a slight increase at 24 h (Fig. S1E, F). In contrast, the viability of SHI-1 cells in the C(A0–0.61+D0.61 and 2) group showed a slight decrease at 48 h, consistent with the Ara-C concentration (Fig. S1G, H). Overall, these results suggest that the limited initial uptake of Ara-C by SHI-1 cells caused a lack of additive or synergistic effects in the combination treatment. Considering the steady and early uptake of DNR by the cells, the uptake of Ara-C in SHI-1 cells was similar between the singular and combination treatments. The [Ara-C]_cell_ values of RHI-1 cells in the A0.64 group were lower than those of SHI-1 cells by 0.7–0.8 times, indicating that low Ara-C uptake may contribute to Ara-C resistance in RHI-1 cells. In contrast, RHI-1 cells in the C(A0.64+D0.61) and C(A0.64+D2) groups had higher [Ara-C]_cell_ values that those exposed to single treatment. Additionally, the [Ara-C]_cell_ values of RHI-1 cells in the C(A0.64+D0.61) and C(A0.64+D2) groups were higher than those of SHI-1 cells by 1.2–1.5 times. Although Ara-C uptake was higher in RHI-1 cells than in SHI-1 cells under the combined treatments, it did not induce additional cytotoxicity in RHI-1 cells, suggesting that RHI-1 cells may have developed Ara-C resistance by inhibiting the mechanism of action of Ara-C.

In contrast, RHI-1 cells in the C(A0.64+D0.61) and C(A0.64+D2) groups had higher [Ara-C]_cell_ values than those exposed to a single treatment. Additionally, the [Ara-C]_cell_ values of RHI-1 cells in the C(A0.64+D0.61) and C(A0.64+D2) groups were higher than those of SHI-1 cells by 1.2–1.5 times. Although Ara-C uptake was higher in RHI cells than in SHI-cells under the combined treatments, it did not induce additional cytotoxicity in RHI-1 cells, suggesting that RHI-1 cells may have developed Ara-C resistance by inhibiting the mechanism of action of Ara-C.

Furthermore, the [DNR]_cell_ values of SHI-1 cells in the D0.61 group were 0.54, 0.62, and 0.64 nmol/10^6^ cells at 1, 24, and 48 h, respectively (Fig. 1D, 1st), whereas those of cells in the D2 group were 0.42, 0.63, and 0.61 nmol/10^6^ cells, respectively (Fig. 1D, 2nd). Additionally, the [DNR]_cell_ values of SHI-1 cells in the C(A0.64+D0.61) group were 0.49, 0.44, and 0.60 nmol/10^6^ at 1, 24, and 48 h, respectively (Fig. 1D, 3rd), whereas those of cells in the C(A0.64+D2) group were 0.44, 0.62, and 0.62 nmol/10^6^ cells, respectively (Fig. 1D, 4th). The [DNR]_cell_ values showed a time-dependent increase in RHI-1 cells in the D0.61, D2, C(A0.64+D0.61), and C(A0.64+D2) groups. Specifically, the [DNR]_cell_ values of RHI-1 cells in the D0.61 group were 0.45, 0.75, and 1.17 nmol/10^6^ cells at 1, 24, and 14 h, respectively (Fig. 1G, 1st), whereas those of cells in the D2 groups were 0.50, 1.30, and 1.64 nmol/10^6^ cells, respectively (Fig. 1G, 2nd). Additionally, the [DNR]_cell_ values of RHI-1 cells in the C(A0.64+D0.61) groups were 0.47, 0.70, and 1.09 nmol/10^6^ cells at 1, 24, and 48 h, respectively (Fig. 1G, 3rd), whereas those of cells in the C(A0.64+D2) group were 0.68, 1.39, and 1.68 nmol/10^6^ cells, respectively (Fig. 1G, 4th).

The [DNR]_cell_ values of SHI-1 cells in the D0.61, D2, C(A0.64+D0.61), and C(A0.64+D2) groups showed a similar trend, ranging from 0.5–0.6 nmol/10^6^ cells. Although the total drug concentration/uptake ([drug]_cell_, sum of [Ara-C]_cells_ and [DNR]_cell_) was higher in the combination treatment groups than in the single treatment groups, DNR had a higher cytotoxicity than Ara-C. Additionally, the [DNR]_cell_ values of RHI-1 cells in the D0.61, D2, C(A0.64+D0.61), and C(A0.64+D2) groups were higher than those of SHI-1 cells by 1.8, 2.7, 1.8, and 2.7 times, respectively. Considering that the drug measurement was performed on living cells, SHI-1 cells may die if the drug concentration exceeds a certain level, at which point the drug concentration cannot be detected. In contrast, RHI-1 cells exhibited tolerance to DNR, indicating that the cells can survive at higher drug concentrations. Overall, these results suggest that Ara-C resistance in RHI-1 may reduce the cytotoxicity of DNR by interrupting the cytotoxic mechanisms of DNR.

Unlike Ara-C, DNR has natural fluorescent properties that enable the visualization of its intracellular distribution. Therefore, we measured intracellular fluorescence intensity of DNR in cells in the D0.61, D2, C(A0.64+D0.61), and C(A0.64+D2) groups after 48 h using confocal fluorescence microscopy. DNR fluorescence intensity was stronger in RHI-1 cells than in SHI-1 cells after each treatment (Fig. 1E, H).

**3. Quantitative proteomic/phosphoproteomic profiling resistance-associated protein signature**

Significantly upregulated or up-phosphorylated proteins contributed to DNR tolerance in RHI-1 cells. To identify these proteins, we compared the proteome of untreated- or treated RHI-1 cells in the D0.61, D2, C(A0.64+D0.61), or C(A0.64+D2) groups with that of untreated SHI-1 cells. The comparison groups were referred to as follows: RHI-1(untreated), RHI-1(0.61 DNR), RHI-1(2 DNR), RHI-1(0.61 Comb), and RHI-1(2 Comb), as shown in Table 2. As mentioned, we utilized the RHI-1(untreated), RHI-1(0.61 Comb), and RHI-1(2 Comb) groups for all subsequent analyses. Differentially expressed proteins (DEPs) and phospho-proteins (phos-DEPs) were identified based on the following criteria: *p* < 0.05 and absolute log2-fold-change $\geq$ 1 (2-fold) (Fig. 2A, Fig. S2C-D, Supplementary Data File 3, 4). In the RHI-1(untreated) group, we identified 12 and 14 upregulated DEPs and phos-DEPs, respectively, which was statistically insufficient for further enrichment analysis. Therefore, we used a cutoff of absolute log 1.5-fold-change for the RHI-1(untreated) group.

**4. Transcriptional changes in RHI-1 cells**

RHI-1 cells was induced via long-term sequential sub-culturing of SHI-1 cells under 0.16, 0.32, and 0.64 μM of Ara-C conditions (19). Therefore, it is believed that the transcriptome of the cells may change under the various concentrations, reflecting adaptation to increasing Ara-C resistance. Accordingly, we performed gene ontology (GO) analysis of differentially expressed genes (upregulated and downregulated; Fig. S3G) in the cells following treatment with 0.16, 0.32, and 0.64 μM of Ara-C from a previously reported study (19).

Upregulated gene ontology biological processes (GOBPs) were associated with ribosome biogenesis, the tricarboxylic acid (TCA) cycle, rRNA processing, and tRNA processing. Additionally, enriched upregulated gene ontology cellular components (GOCCs) category were associated with mitochondria, nuclei, and mitochondrial nucleoids. According to the Warburg effect, most cancer cells, including AML, predominantly process carbohydrates and generate energy through glycolysis, even in the presence of oxygen (20). Upregulated genes were highly associated with mitochondrial metabolism, suggesting that mitochondrial metabolism may be associated with Ara-C resistance in RHI-1 cells. Although some downregulated genes were enriched in the GOBP and GOCC categories, they were mostly associated with homeostatic processes and not drug resistance.

Ara-C resistance involves the deletion of the DCK gene to suppress the conversion of Ara-C to its active form. Additionally, aberration of SLC29A1 and SLC28A1/SLC28A3 genes, which inhibit Ara-C uptake, leads to Ara-C resistance. Moreover, the amplification of ABCC10/11 gene, which effluxes Ara-C, results in Ara-C resistance (12). Among these genes, only DCK expression showed a gradual decrease with increasing Ara-C resistance (Table S4). Overall, these results suggest that downregulation of DCK-mediated Ara-C resistance is highly associated with mitochondrial metabolism in RHI-1 cells.

**Supplemental Data Items**

**Supplementary Table 1.** The viability of SHI-1 cells at 48h

|  | | Concentration of Ara-C | | | | | | | |
| --- | --- | --- | --- | --- | --- | --- | --- | --- | --- |
|  | | 0 | | 0.16 | | 0.32 | | 0.64 | |
|  |  | mean | S.D. | mean | S.D. | mean | S.D. | mean | S.D. |
| Concentration of DNR | 0 | 100 | ±9.4 | 73 | ±7.3 | 67 | ±5.7 | 55 | ±4.4 |
|  | 0.08 | 60 | ±5.2 | 56 | ±0.9 | 48 | ±2.9 | 44 | ±3 |
|  | 0.16 | 24 | ±9.6 | 26 | ±11.5 | 25 | ±12.6 | 24 | ±10.4 |
|  | 0.2 | 23 | ±4.5 | 22 | ±4.2 | 21 | ±4.1 | 21 | ±3.5 |
|  | 0.3 | 15 | ±4.0 | 15 | ±3.5 | 14 | ±3.3 | 14 | ±3.4 |
|  | 0.61 | 12 | ±3.0 | 11 | ±2.8 | 11 | ±2.5 | 11 | ±2.4 |
|  | 2 | 11 | ±2.6 | 11 | ±2.7 | 11 | ±2.7 | 11 | ±2.7 |

**Supplementary Table 2.** The viability of RHI-1 cells at 48h

|  | | **Concentration of Ara-C** | | | | | | | |
| --- | --- | --- | --- | --- | --- | --- | --- | --- | --- |
|  | | 0 | | 0.16 | | 0.32 | | 0.64 | |
|  |  | mean | S.D. | mean | S.D. | mean | S.D. | mean | S.D. |
| **Concentration of DNR** | 0 | 100 | ±0 | 100 | ±0.8 | 101 | ±1.1 | 101 | ±2 |
|  | 0.08 | 78 | ±13.9 | 78 | ±13.4 | 78 | ±15.3 | 76 | ±8.5 |
|  | 0.16 | 72 | ±9.6 | 70 | ±7.3 | 73 | ±9.3 | 70 | ±7.7 |
|  | 0.2 | 71 | ±9.8 | 70 | ±8.1 | 69 | ±10 | 74 | ±9.5 |
|  | 0.3 | 60 | ±6.7 | 60 | ±6.8 | 60 | ±7.4 | 62 | ±7.2 |
|  | 0.61 | 46 | ±5.0 | 46 | ±5.8 | 47 | ±4.7 | 47 | ±4.4 |
|  | 2 | 14 | ±4.3 | 14 | ±3.9 | 14 | ±3.8 | 15 | ±4.7 |

|  | | | [Ara-C]_cell_ | | | | | [DNR]_cell_ | | | | | |
| --- | --- | --- | --- | --- | --- | --- | --- | --- | --- | --- | --- | --- | --- |
| treatment | Time (h) | nmol/10^6^ cells | | | | | ratio | nmol/10^6^ cells | | | | ratio | |
|  |  | SHI-1 | | | RHI-1 | | RHI-1  /SHI-1 | SHI-1 | | RHI-1 | | | RHI-1  /SHI-1 |
| A0.64 | 1 | 0.35 | | ±0.061 | 0.27 | ±0.036 | 0.8 | - | - | - | - | | - |
|  | 24 | 0.37 | | ±0.07 | 0.27 | ±0.042 | 0.7 | - | - | - | - | | - |
|  | 48 | 0.38 | | ±0.074 | 0.29 | ±0.075 | 0.8 | - | - | - | - | | - |
| D0.61 | 1 | - | | - | - | - | - | 0.54 | ±0.141 | 0.45 | ±0.115 | | 0.8 |
|  | 24 | - | | - | - | - | - | 0.62 | ±0.162 | 0.75 | ±0.236 | | 1.2 |
|  | 48 | - | | - | - | - | - | 0.64 | ±0.167 | 1.17 | ±0.305 | | 1.8 |
| D2 | 1 | - | | - | - | - | - | 0.42 | ±0.11 | 0.50 | ±0.132 | | 1.2 |
|  | 24 | - | | - | - | - | - | 0.63 | ±0.165 | 1.30 | ±0.451 | | 2.1 |
|  | 48 | - | | - | - | - | - | 0.61 | ±0.158 | 1.64 | ±0.576 | | 2.7 |
| C(A0.64+D0.61) | 1 | 0.28 | | ±0.03 | 0.33 | ±0.056 | 1.2 | 0.49 | ±0.104 | 0.47 | ±0.077 | | 1.0 |
|  | 24 | 0.29 | | ±0.057 | 0.40 | ±0.036 | 1.4 | 0.44 | ±0.116 | 0.70 | ±0.183 | | 1.6 |
|  | 48 | 0.37 | | ±0.076 | 0.44 | ±0.083 | 1.2 | 0.60 | ±0.157 | 1.09 | ±0.248 | | 1.8 |
| C(A0.64+D2) | 1 | 0.22 | | ±0.023 | 0.34 | ±0.048 | 1.5 | 0.44 | ±0.114 | 0.68 | ±0.342 | | 1.5 |
|  | 24 | 0.34 | | ±0.043 | 0.50 | ±0.098 | 1.5 | 0.62 | ±0.163 | 1.39 | ±0.381 | | 2.2 |
|  | 48 | 0.37 | | ±0.081 | 0.52 | ±0.115 | 1.4 | 0.62 | ±0.162 | 1.68 | ±0.409 | | 2.7 |

**Supplementary Table 3.** [Drug]_cell_ in SHI-1The viability of RHI-1 cells at 48h

**Supplementary Table 4.** log2(Fold-change) of Ara-C resistance-related genes.

|  | Normalized expression level | | | |
| --- | --- | --- | --- | --- |
| Gene | SHI-1 parental | 160 Ara-C  Resistance | 320 Ara-C  Resistance | 640 Ara-C  Resistance |
| SLC29A1 | 1.000 | 1.025 | 1.015 | 1.003 |
| SLC28A3 | 1.000 | 0.953 | 0.931 | 0.938 |
| SLC28A1 | 1.000 | 0.989 | 1.000 | 1.007 |
| ABCC10 | 1.000 | 0.977 | 0.995 | 0.983 |
| ABCC11 | 1.000 | 0.997 | 1.013 | 0.995 |
| DCK | 1.000 | 0.950 | 0.935 | 0.843 |

**

**

**Supplementary Table 5. C**linical characteristics of AML patients

| *Patient identifier* | *Result of Bone marrow biopsy and aspiration* | *Treatment response* |
| --- | --- | --- |
| AML-P1 | Acute Myeloid Leukemia with KMT2A::AFDN Rearrangement, Hypercellular Marrow with Blasts plus Promonocytes 73.9% | Responsive |
| AML-P2 | Acute Myeloid Leukemia without maturation,  Hypocellular Marrow with Blasts 49.2%. | Responsive |
| AML-P3 | Acute myeloid leukemia, myelodysplasia-related (AML-MR) | Responsive |
| R/R-P1 | Treated Acute Myeloid Leukemia, Myelodysplasia-Related (AML-MR) (r/o Post Cytotoxic Therapy), s/p Induction Tx D56, in Persistence Normocellular Marrow with Blasts 29.0% and Megakaryocytic Dysplasia | Resistant |
| R/R-P2 | Treated Acute Myeloid Leukemia with NPM1 Mutation (type A mutation), s/p Induction(cytarabine, daunorubicin) D48, in Persistence Hypercellular Marrow with Blasts 89.1% | Resistant |
| R/R-P3 | Treated Acute Myeloid Leukemia with KMT2A::MLLT10 Rearrangement, D#29 1L1C Hi Daunorubicin/Standard Cytarabine, in Persistence  Slightly Hypercellular Marrow with Blasts 54.0% | Resistant |


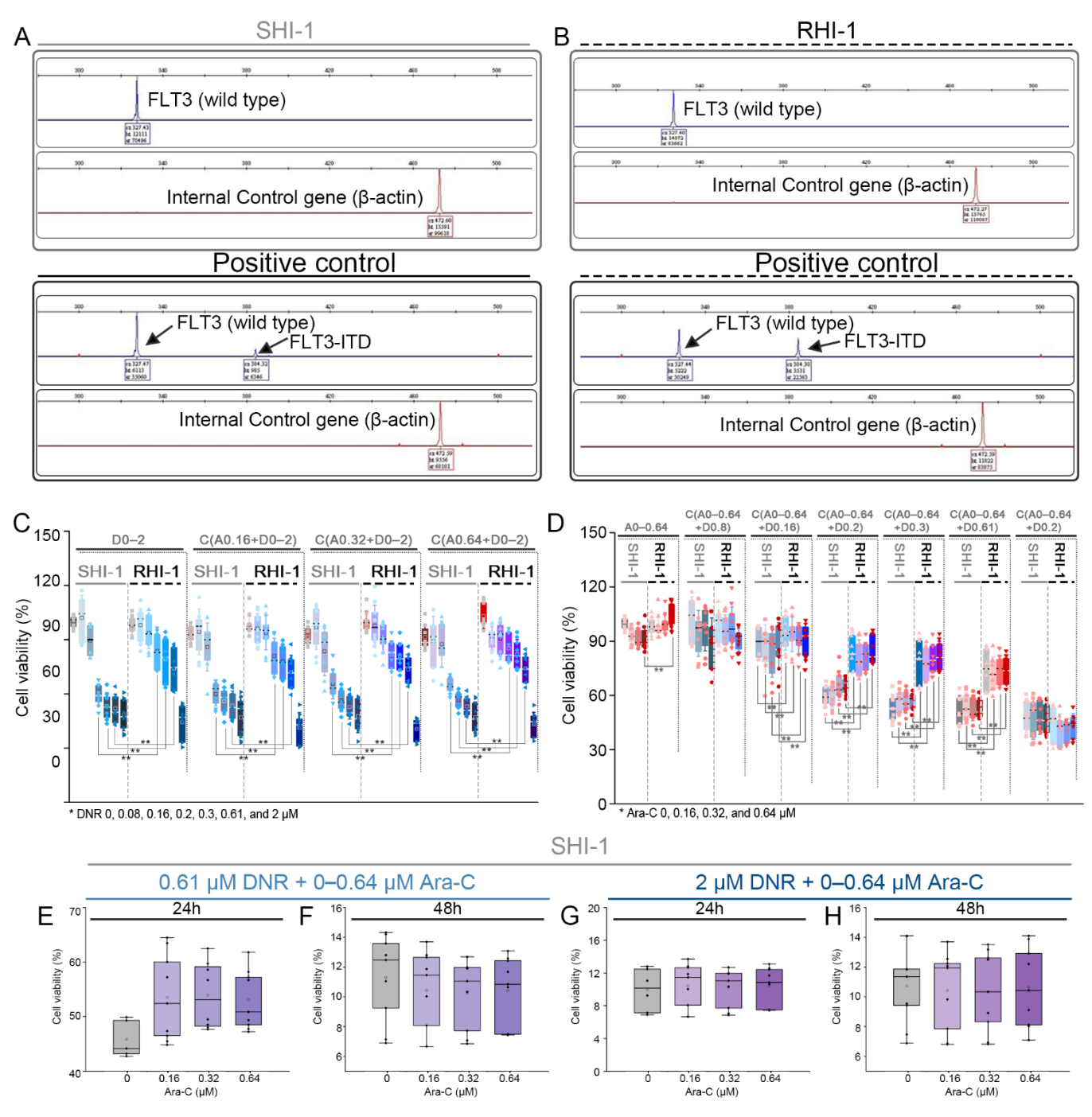


Supplementary Figure 1. FLT3-ITD Mutation Analysis and Cell Viability Responses to Ara-C and DNR Combinations in SHI-1 and RHI-1 Cell Lines (A, B) Results of FLT3-ITD mutation fragment analysis. (C-D) Results of cell viability in SHI-1 and RHI-1 cells at 24 h. (C) Box plots showing cell viability obtained under D0–D2, C(A0.16+D0–2), C(A0.32+D0–2), and C(A0.64+D0–2). (D) Box plots showing cell viability obtained from A0–A0.64, C(A0–A0.64+D0.08), C(A0–A0.64+D0.16), C(A0–A0.64+D0.2), C(A0–A0.64+D0.3), C(A0–A0.64+D0.61), and C(A0–A0.64+D0.2) treated cells. (E-H) Cell viability of 0.61 and 2 μM DNR treated SHI-1 and RHI-1 with various Ara-C concentrations. ∗∗p < 0.01.


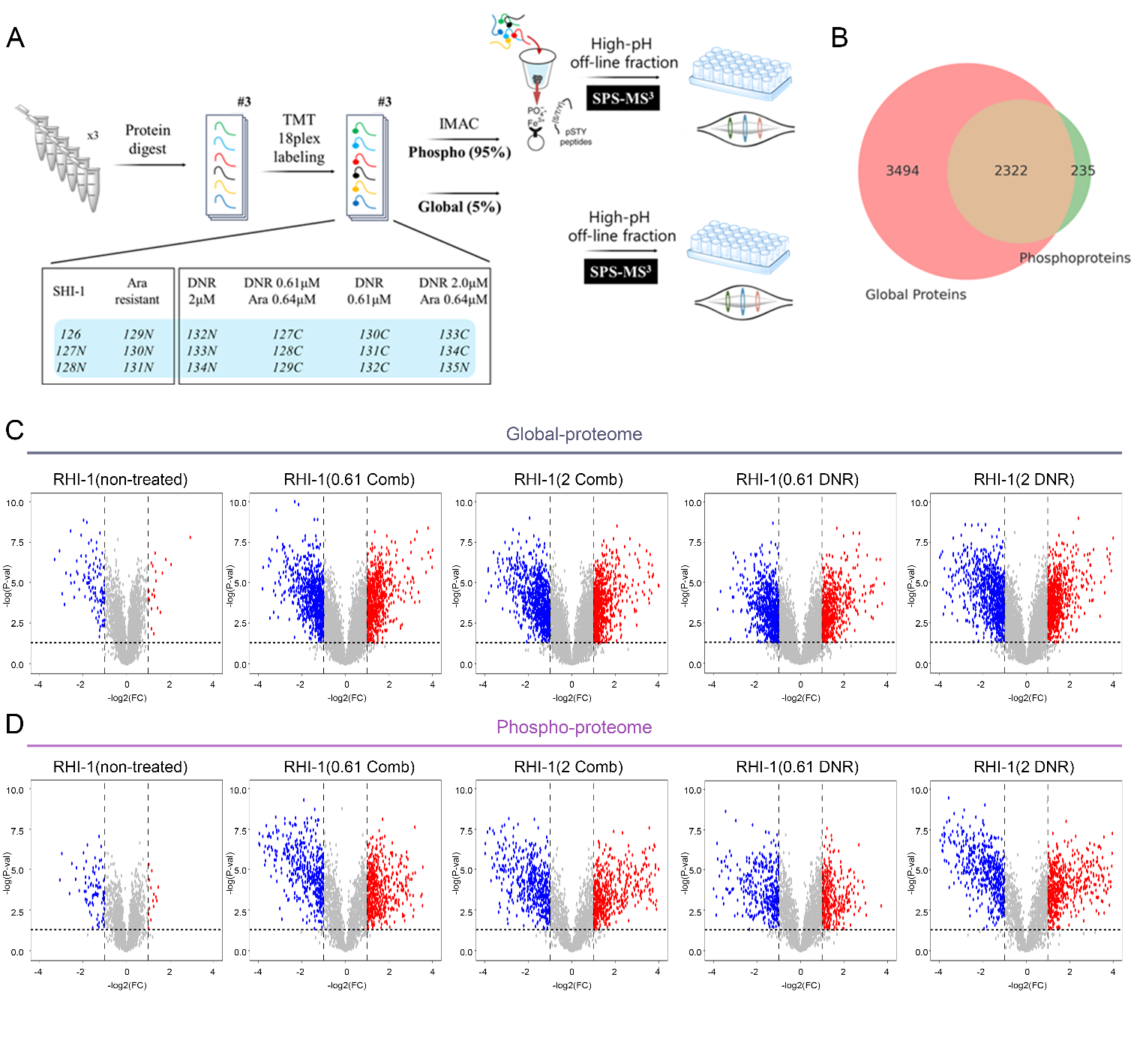


**Supplementary Figure** **2. Global proteome and phosphoproteome MS3 profiling of AML cells treated with DNR or Ara.** (A) Schematic representation of the experimental procedure for quantitative MS3 proteomic analysis. All samples were subjected to digestion and 18plex TMT-labeling as a common process. The 5% of the TMT-labeled peptides were used for global proteomics, while the remaining 95% were used for phosphoproteomics after phosphopeptide enrichment through IMAC. After high-pH fraction, the resulting phosphor/global fractions were separated on-line by nanoflow liquid chromatography an analyzed by high-resolution tandem mass spectrometry. (B) The numbers of proteins identified from global and phosphoprotein. (C-D) Volcano plot of proteomics and phosphoproteomics data. Up-regulated proteins are marked with red, and down-regulated proteins are marked with blue. Volcano plots are obtained from three independent global and phosphoproteomics experiments.

**
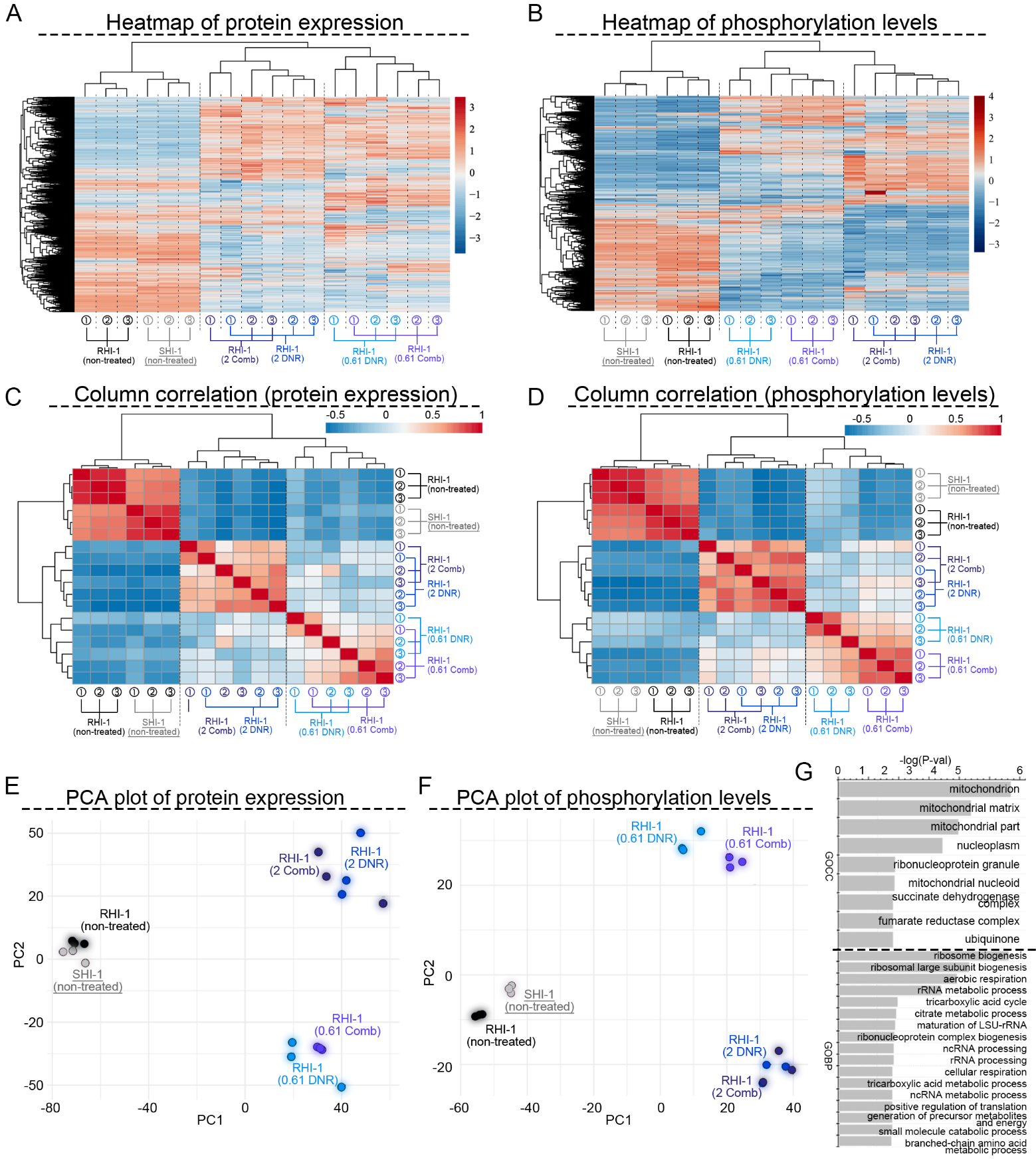
**

Supplementary Figure 3. Sample correlation analysis (A, B) Heatmap for protein expression (A) and phosphorylation levels (B). (C, D) Heatmap for column correlation using protein expression (C) and phosphorylation levels (D) (E, F) Principal component analysis (PCA) using protein log2(fold-change) values. The cumulative proportion of the first principal component (PC1, x-axis) and the second principal component (PC2, y-axis) accounts for 94% in both global and phospho-proteomics analyses. (G) GO enrichment analysis using transcriptome whose expressions increased in RHI-1 cells compared to SHI-1 cells. Bar charts with the enriched gene ontology cellular components (GOCCs) and Gene Ontology biological processes (GOBPs).


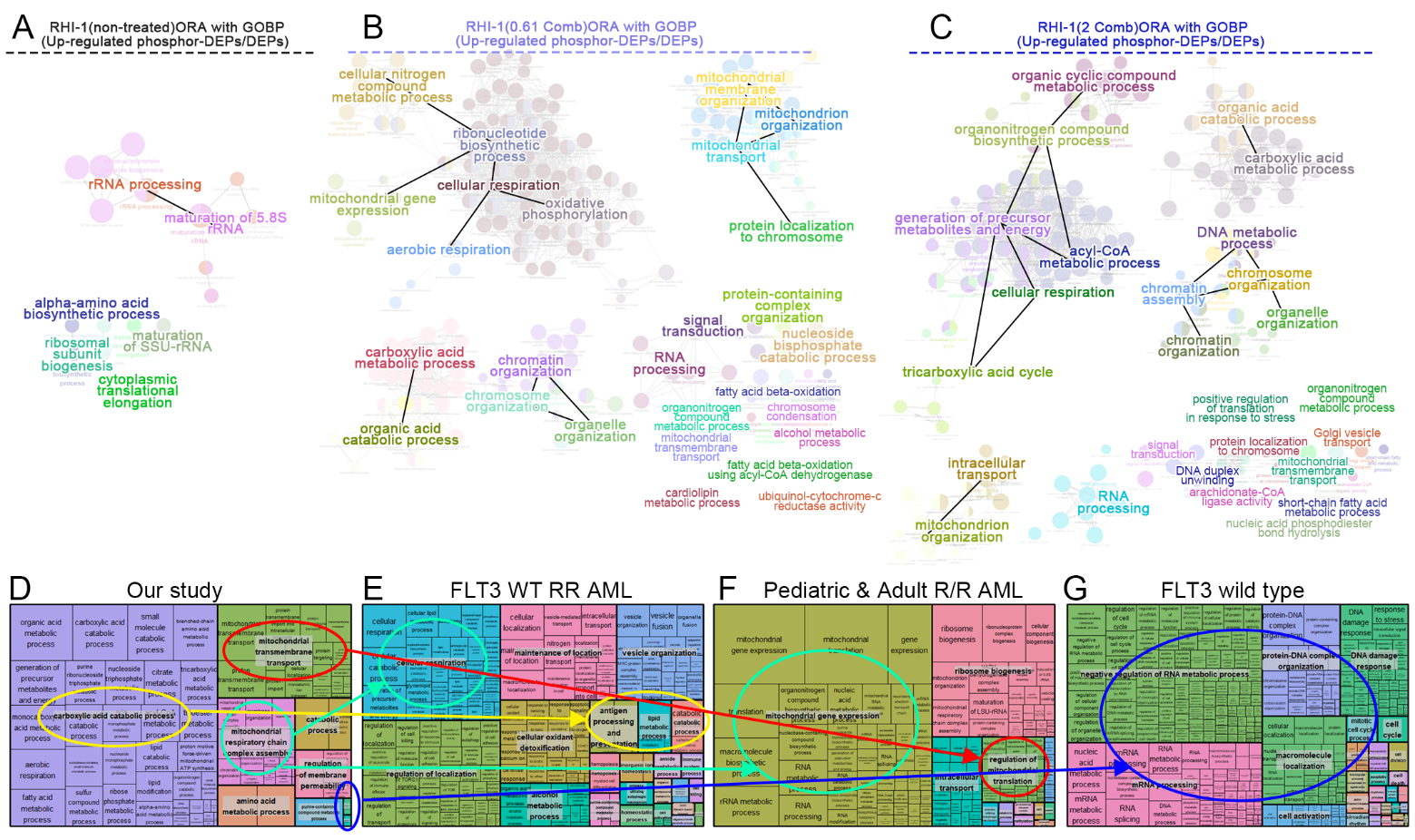


Supplementary Figure 4. Gene ontology analysis and cluster network (A-C) Gene ontology biological pathway cluster networks—data derived from three independent experiments. (D-G) GO-analysis of relapse-associated significantly altered proteins in wild type R/R AML, pediatric/Adult R/R AML, and FLT3 wild type vs FLT3 mutated type AML at previously study.
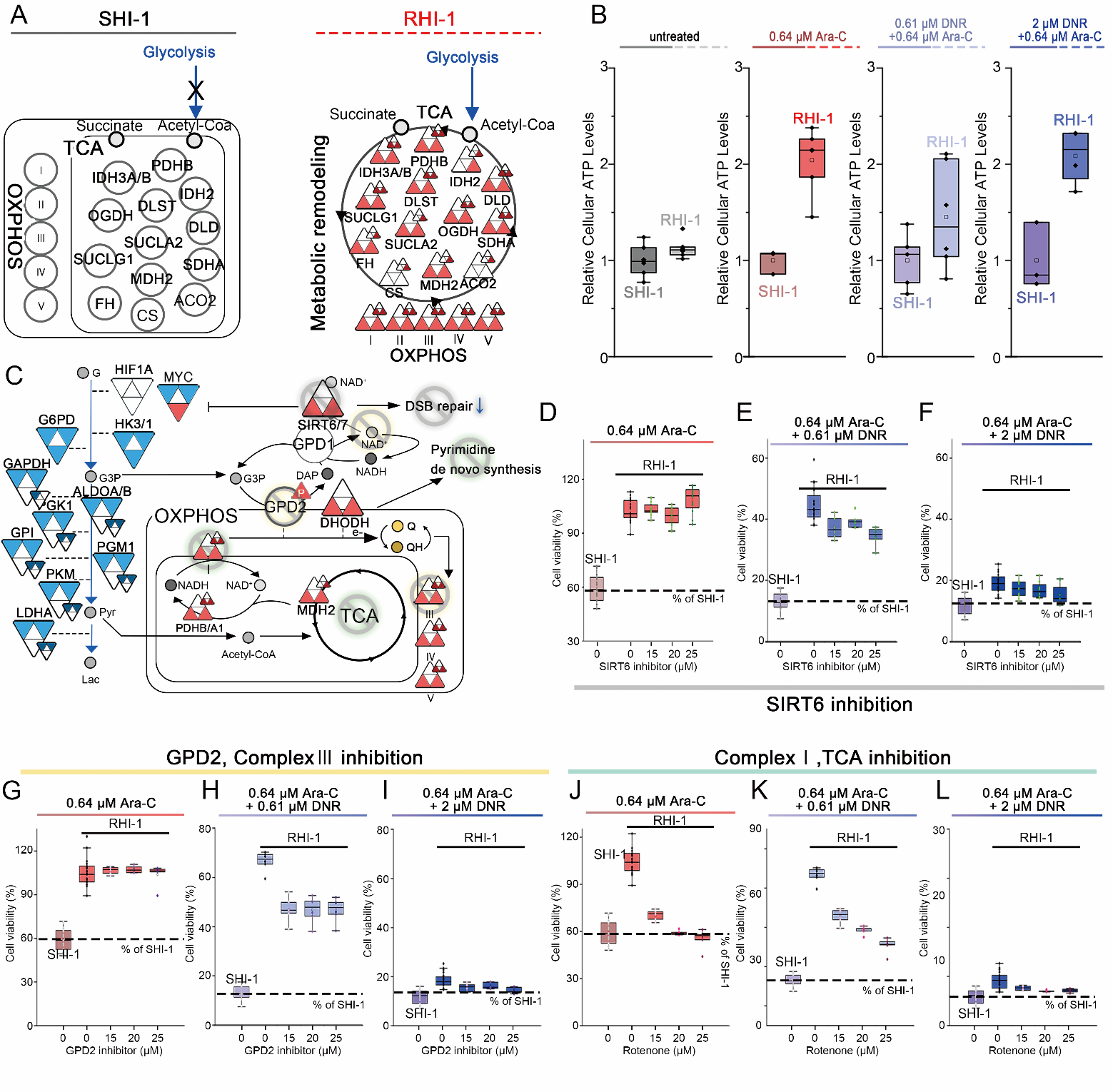


Supplementary Figure 5. Metabolic Remodeling in SHI-1 and RHI-1 Cells Under Ara-C and DNR Treatment (A) Schematic illustration of a metabolic process in SHI-1 and RHI-1 cells. (B) Cellular ATP levels in SHI-1 and RHI-1 cells under drug treatment.


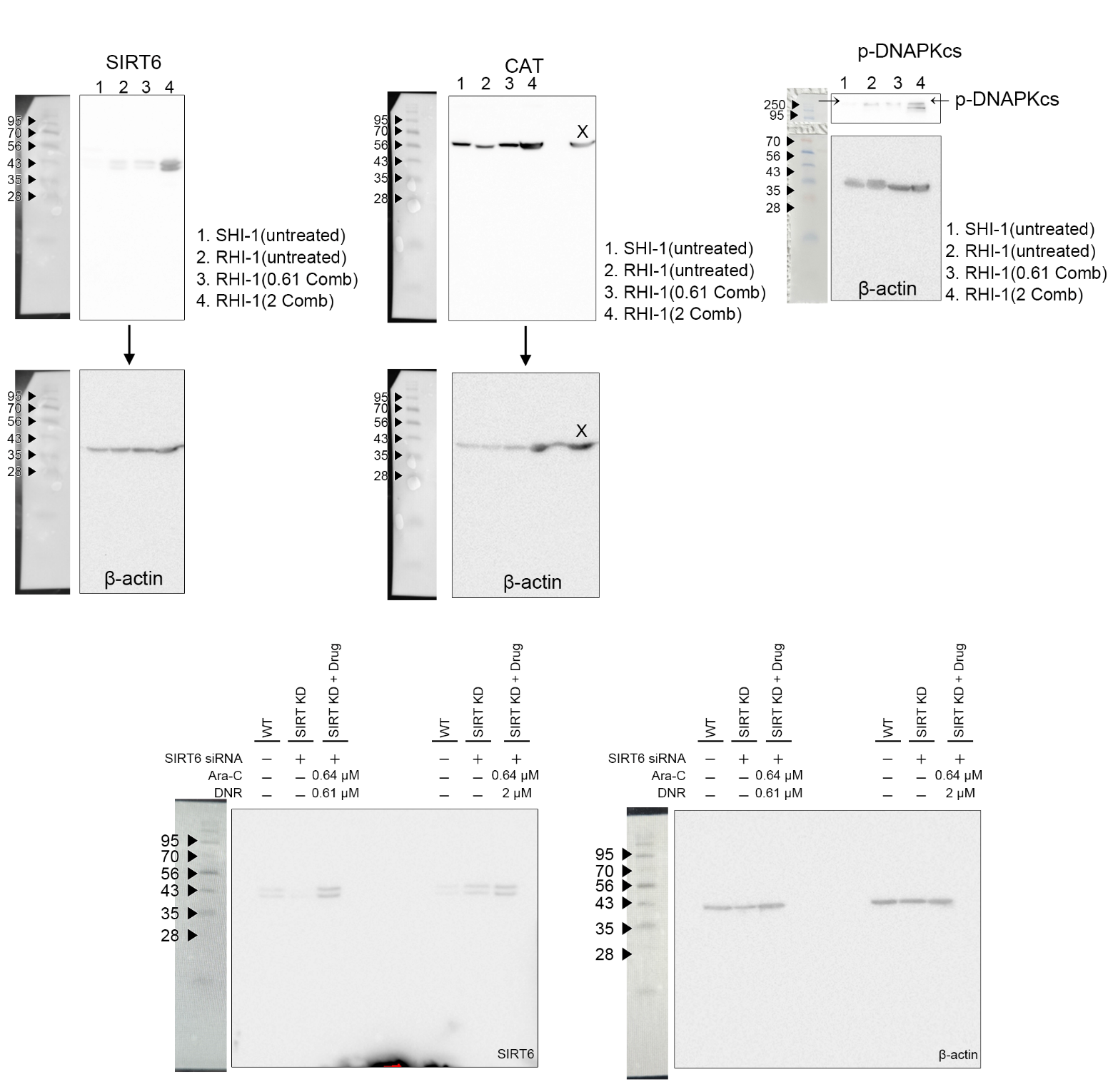


Supplementary Figure 6. Original western blots used in Figure 2.
