# Supplementary material for: Mechanisms of chemotherapy failure in refractory/relapsed acute myeloid leukemia: the role of cytarabine resistance and mitochondrial metabolism
Source: Cell Death Dis. 2025 Apr 23;16(1):331. doi: 10.1038/s41419-025-07653-6 (PMC12019594; doi:10.1038/s41419-025-07653-6)
Supplement: Supplementary file 2 — Original Western blot [file 41419_2025_7653_MOESM2_ESM.docx]

(uncropped) western blot

Mechanisms of chemotherapy failure in refractory/relapsed acute myeloid leukemia: The role of cytarabine resistance and mitochondrial metabolism

Soo Yeon Chae^1,2,‡^, Se-Young Jang^3,‡^, Jinhui Kim^1^, Sehyun Hwang^1,2^, Disha Malani^6,7^, Olli Kallioniemi^7,8^, Seung Gyu Yun^5,*^, Jong-Seo Kim^3,4,*^, Hugh I. Kim^1,2,9,*^

^1^ Department of Chemistry, Korea University, Seoul, 02841, Republic of Korea

^2^ Center for Proteogenome Research, Korea University, Seoul, 02841, Republic of Korea

^3^ School of Biological Sciences, Seoul National University, Seoul, 08826, Korea

^4^ Center for RNA Research, Institute of Basic Science, Seoul National University, Seoul, 08826, Korea

^5^ Department of Laboratory Medicine, Korea University College of Medicine, Seoul, Korea.

^6^ Department of Medical Oncology, Dana-Farber Cancer Institute, Boston, Massachusetts.

^7^ Institute for Molecular Medicine Finland, FIMM, University of Helsinki, Helsinki, Finland

^8^ Science for Life Laboratory, Department of Oncology and Pathology, Karolinska Institutet, Solna, Sweden

^9^ Lead Contact

‡ These authors contributed equally.

*Correspondence: [koryun@korea.ac.kr](mailto:koryun@korea.ac.kr), [jongseokim@snu.ac.kr](mailto:jongseokim@snu.ac.kr), [hughkim@korea.ac.kr](mailto:hughkim@korea.ac.kr)


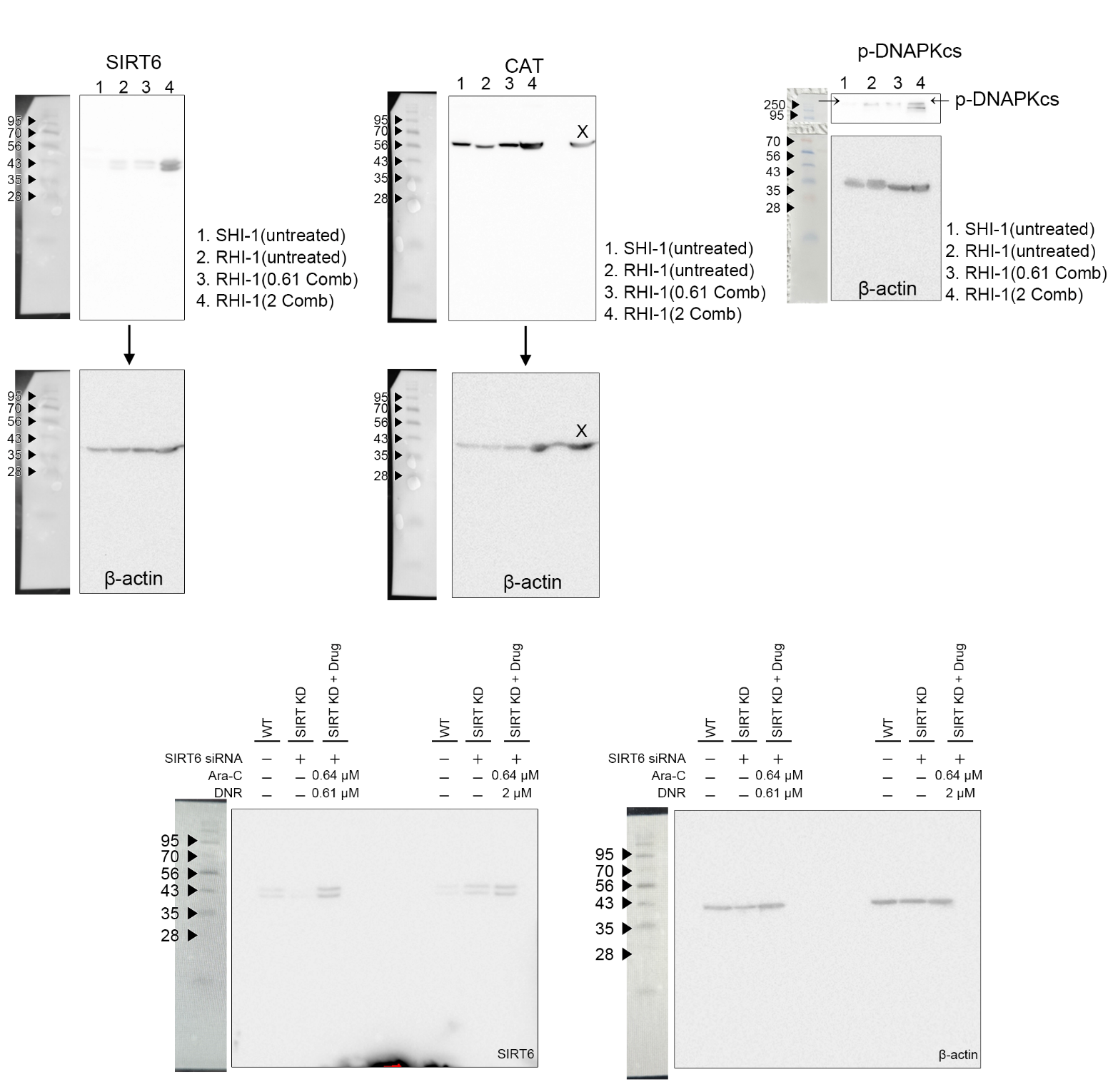


Original western blots used in Figure 2.
